# Short- and long-term exposure to ambient air pollution and greenness in relation to pulmonary tuberculosis incidence

**DOI:** 10.1038/s41598-025-11465-1

**Published:** 2025-07-15

**Authors:** Dongmiao Yuan, Bo Xie, Zhe Pang, Kui Liu, Bin Chen

**Affiliations:** 1 School of Urban Design, Wuhan University, Wuhan, 430072 China; 2 Zhejiang Provincial Center for Disease Control and Prevention, Hangzhou, 310051 China

**Keywords:** Pulmonary tuberculosis, Air pollutants, Lag effects, Greenness exposure, Modification effects, Environmental impact, Diseases, Risk factors

## Abstract

Epidemiological studies have found inconsistent relationships between air pollutants and the risk of pulmonary tuberculosis (PTB), possibly due to variations in exposure windows and limited attention to environmental modifiers such as greenness. However, few studies has systematically examined how short- and long-term exposure to air pollution may differentially impact PTB risk, and how greenness may modify these associations. We utilized comprehensive data, including daily PTB incidence, air pollutants, meteorological data, and the normalized difference vegetation index (NDVI) from Zhejiang Province, China, spanning from 2013 to 2019. A distributed lag nonlinear model (DLNM) was employed to examine the relationships between air pollution and PTB incidence by county, and a meta-analysis was conducted to aggregate county-specific estimates. In the single-pollutant model, the lag-specific excess risk (ER) of PTB was 0.7% (95% CI 0.05%, 1.4%, 13-week lag) for each 0.1 mg/m^3^ increase in carbon monoxide (CO). For each 10 µg/m^3^ increase in the combined oxidant capacity (O_X_), the lowest risk was a 0.9% decrease (95% CI −1.5%, −0.3%, 16-week lag). For each 10 µg/m^3^ increase in particulate matter 2.5 (PM_2.5_), the highest risk was a 1.7% increase (95% CI 0.8%, 2.6%, 19-week lag). Conversely, each 10 µg/m^3^ increase in sulfur dioxide (SO_2_) showed a dual association with PTB incidence, encompassing a short-term negative correlation and a long-term positive correlation. Furthermore, the associations between CO and PM_2.5_ and PTB incidence were more pronounced in the male and working-age subgroups, whereas the associations with SO_2_ were more significant in the female and elderly subgroups. Additionally, we observed that greenness negatively modified the relationship between short- and long-term exposure to O_X_ and PTB incidence. Our findings revealed significant long-term lagged effects of CO, O_X_, and PM_2.5_ on PTB incidence, as well as short- and long-term lagged effects of SO_2_. Furthermore, greenness was identified as a modifier of the association between O_X_ and PTB incidence at various lag times.

**Supplementary Information:**

The online version contains supplementary material available at 10.1038/s41598-025-11465-1.

## Introduction

Pulmonary tuberculosis (PTB), a form of tuberculosis (TB) caused by *Mycobacterium tuberculosis* (MTB), is a contagious wasting disease that mainly affects the lungs^1^. As a result of the initial dormancy of MTB, over 90% of infected individuals remain asymptomatic. However, as the immune system weakens, the disease may eventually progress to active PTB^2,3^. According to the World Health Organization, approximately 7.5 million newly reported TB cases and 1.3 million TB-related deaths were documented globally in 2022. Notably, about 87% of global TB cases occur in 30 high-burden developing countries, with the top eight nations, including China, accounting for two-thirds of the overall burden^4^.

The relationship between PTB risk and environmental factors, including air pollutants and meteorological conditions, is well-established^5–9^. Since MTB is transmitted via airborne particles, numerous studies have examined the influence of short- and long-term exposure to air pollutants on PTB risk, accounting for potential lagged effects in their analyses^10–13^. However, significant heterogeneity has been observed in these associations. For instance, the relationship between pollutant exposure and the risk of PTB varies depending on the duration of exposure. A study in Wulumuqi, China, revealed a positive correlation between long-term exposure to carbon monoxide (CO), ozone (O_3_), particulate matter 2.5 (PM_2.5_), and particulate matter 10 (PM_10_) and PTB risk^14–16^. In contrast, a retrospective study in Spain found no significant associations between short-term exposure to CO, PM_2.5_, and PM_10_ and PTB incidence^17^. Additionally, several studies have shown that while sulfur dioxide (SO_2_) exhibits short-term protective effects^17–19, ^it also exerts harmful long-term effects on PTB risk^20–22^. The lagged effects of air pollution on PTB risk also varied depending on specific and cumulative lag times. For example, a study in Beijing, Tianjin, Hebei, China, found that for each 10 µg/m^3^ increase in PM_2.5_, the maximum lag-specific risk was 1.011 (at a 3-month lag) and cumulative relative risk (RR) was 1.042 (at a 5-month lag), respectively^10^. Previous studies have suggested that these heterogeneous associations may arise from variations in air pollutant concentrations, geographical environments, population characteristics, and exposure assessment methods^23–25^. However, few studies have simultaneously investigated both the specific and cumulative lagged effects of air pollutant exposure in relation to PTB incidence.

Greenness is recognized for its health benefits through various pathways, such as reducing exposure to air pollution, promoting mental health, and encouraging physical activity^26^. Previous studies have demonstrated that greenness can modify the associations between long-term PM_2.5_ exposure and PTB risk. For instance, a population-based retrospective study found that higher levels of greenness may mitigate the adverse effects of long-term PM_2.5_ exposure on the risk of PTB retreatment^27,28^. A nationwide analysis revealed that greenness modified the relationships between long-term PM_2.5_ exposure and the incidence of smear-positive PTB^29^. Additionally, air pollutants exert both chronic and acute effects on PTB risk. For instance, a time-series study demonstrated that NO_2_ had an acute harmful effect on TB risk, likely due to its significant role in the formation of photochemical smog, which adversely affected pulmonary health^30^. However, most existing studies primarily focus on investigating the modifying effects of greenness on the associations between long-term air pollution exposure and PTB risk^28,31, ^while overlooking its potential influence on association between short-term air pollution exposure and PTB risk.

This study utilized a two-stage analytic approach to conduct a seven-year time-series analysis in Zhejiang Province spanning from January 1, 2013, to December 31, 2019. The aim was to explore the association between short- and long-term exposure to air pollutants (CO, O_X_, PM_2.5_, and SO_2_) and PTB incidence, while also evaluating greenness as a potential effect modifier.

## Materials and methods

### PTB cases

**Fig. 1:**
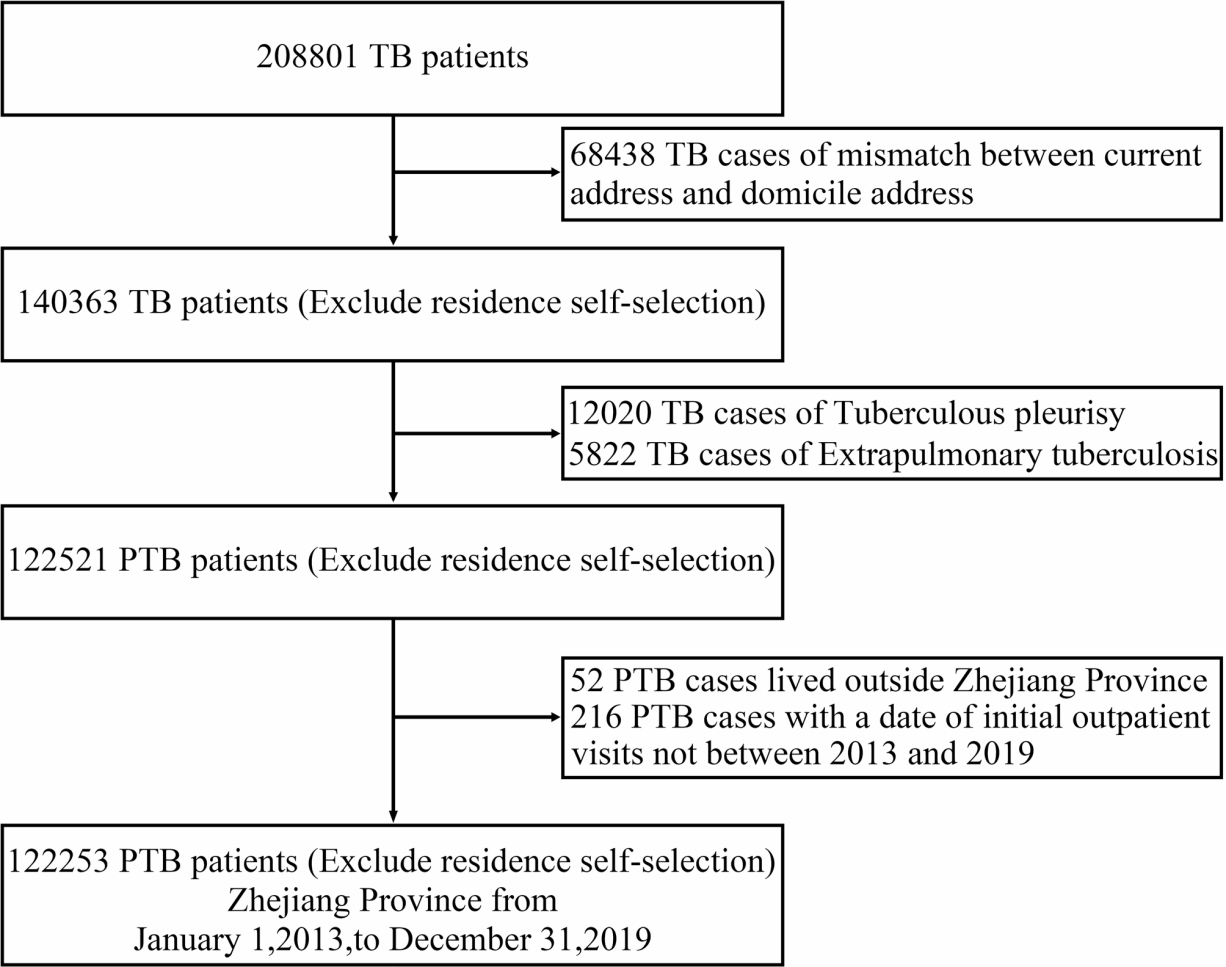
PTB patient enrollment flow chart.

PTB is classified as Class B for infectious disease in China^32^. According to national tuberculosis control guidelines, medical institutions are required to report diagnosed PTB cases within 24 hours using the infectious disease reporting system^33^. All suspected PTB cases were diagnosed based on the criteria outlined in TB diagnostic standards (WS 288-2008 and WS 288-2017), which include chest imaging, etiological examinations, assessments of the patient’s clinical symptoms, epidemiological history, and other relevant diagnostics (http://www.nhc.gov.cn/). Our study collected daily PTB case data from the Zhejiang Provincial Centre for Disease Control and Prevention (Zhejiang CDC) across 90 counties in Zhejiang Province from January 1, 2013, to December 31, 2019. We excluded 68,438 PTB cases due to mismatches between current and residential addresses, 12,020 cases of tuberculous pleurisy, 5,822 cases of extrapulmonary tuberculosis, 52 cases from outside Zhejiang Province, and 216 cases with initial outpatient visit dates outside the study timeframe (Fig. 1). Ultimately, the date of the initial outpatient visit for PTB was used as the time index for the weekly time-series analyses.

### Measurement of ambient air pollutants and meteorological factors

First, the daily average concentration raster data for PM_2.5_, PM_10_, NO_2_, SO_2_, O_3_, and CO, at a spatial resolution of 1 km×1 km, were obtained from the National Earth System Science Data Center in Zhejiang Province (https://geodata.nnu.edu.cn/featured_data.html). Second, daily meteorological data for Zhejiang Province, including total precipitation (TP), average temperature (AT), relative humidity (RH), sunshine duration (SD), and wind speed (WS), were obtained from the China Meteorological Science Data Sharing Service System (http://data.cma.cn/). As the meteorological data are derived from observations at weather monitoring stations, we initially applied the inverse distance weighting (IDW) interpolation method to generate daily average raster data. Subsequently, using these daily raster data, we calculated weekly averages of air pollutants and meteorological factors for each county throughout the study period. Additionally, the combined oxidant capacity (O_X_) was calculated as an environmental factor using the formula proposed by Bratsch^34^. Given the extremely low missing rates for both air pollution and meteorological variables (each below 0.3%), no imputation was performed for the missing values.

### Greenness exposure

Greenness was assessed using the normalized difference vegetation index (NDVI), which was sourced from satellite images through the Moderate Resolution Imaging Spectroradiometer (MODIS). The NDVI scale ranges from − 1 to 1, where negative values reflect features such as water or clouds, a value of 0 represent bare earth or rock, and positive values indicate healthy vegetation, with higher values indicating greater greenness. Monthly average NDVI values for each county in Zhejiang Province were calculated from 16-day data with a 1-km resolution. In accordance with the stratification method used in previous studies^29,35^and considering the clustered distribution of NDVI values, median stratification was adopted to ensure balanced sample sizes, thereby enhancing statistical comparability and model robustness. Specifically, counties in Zhejiang Province were categorized into high- and low-NDVI groups based on the median NDVI value.

### Statistical analysis

To characterize the distribution characteristics of PTB incidence, as well as ambient air pollutants, meteorological factors, and greenness, we calculated the following statistical metrics: mean, standard deviation (SD), minimum (min), maximum (max), first quartile (25th percentile, P25), second quartile (50th percentile, P50), third quartile (75th percentile, P75), and interquartile range (IQR). Additionally, a time series plot was generated. Spearman’s rank correlation coefficients were utilized to evaluate the associations between these variables, while Cohen’s kappa was employed to avoid multicollinearity^36^.

To estimate the effects of short- and long-term exposure to ambient air pollutants (CO, O_X_, PM_10_, PM_2.5_, and SO_2_) on PTB incidence, we employed a standardized two-phase analytical approach^29,37^.

First, to account for over-dispersion in the data, a distributed lag nonlinear model (DLNM) with a quasi-Poisson distribution was utilized to evaluate the lag effects of ambient air pollutants on PTB incidence at both specific and cumulative lag times for each county. We also performed a subgroup analysis by dividing the PTB data into male, female, working-age, and elderly subgroups. Short-term exposure to air pollutants was defined as lasting between 0 and 4 weeks, while long-term exposure extended from 4 to 26 weeks^38^. The final model is described below:

$${\text{Log }}\left[ {{\text{E }}\left( {{{\text{Y}}_{{\text{it}}}}} \right)} \right]{\text{ }}={\text{ }}{{\text{a}}_{\text{i}}}+{\text{ }}{{\text{W}}_{{\text{x}},{\text{t}}\eta }}+{\text{ns }}\left( {{\text{tim}}{{\text{e}}_{\text{i}}},{\text{ 6}}} \right)+\sum {\text{ ns }}\left( {{\text{W}},{\text{ 3}}} \right)$$

where Y_it_ refers to PTB incidence in county *i* during week t, and a_i_ denotes the model intercept. Taking into account the lagged effects of air pollutants observed in studies spanning from 3 to 6 months^29,39^, the longest lag time was set at 26 weeks based on the Akaike Information Criterion for quasi-Poisson models (Q-AIC). Based on previous research, the cross-basis matrix for air pollution in the distributed lag nonlinear model is generally constructed using a cubic spline function with three degrees of freedom for both the exposure-response and exposure-lag dimensions^3^, denoted as *W*_*x, tη*_. Long-term trends and cyclical variations are commonly modeled using a cubic spline function with six degrees of freedom^1,40,41^, expressed as ns *(time*_*i*_, *6)*, where *Time*_*i*_ represents the “week” *(week = 1*,* 2*,* 3*,* …*,* 366)*. Other covariates, presented as weekly averages, are generally represented by a cubic spline function with three degrees of freedom^13,29,37^, denoted as*∑ ns (W*,* 3)*. *W* includes AT, WS, RH, SD, and TP. In the case of a multi-pollutant model, *W* also incorporates additional air pollutants not included in the cross-basis matrix. To ensure the robustness of parameter selection, we used the QAIC method to assess the degrees of freedom for *W*_*x, tη*_, *Time*_*i*_, and *W*, confirming their appropriateness.

Second, a meta-analysis was conducted to aggregate all county-specific effect estimates using the same model and evaluate the associations between air pollutants and PTB incidence by employing the restricted maximum likelihood method. Additionally, we used NDVI for a stratified analysis to investigate the modifying effect of greenness on these associations.

Excess risk (ER) and 95% confidence intervals (CIs) were estimated at both specific and cumulative lag windows, using the median air pollution concentration as the reference. Although the DLNM captures the non-linear relationship through cubic spline functions, we focused on reporting key point estimates, such as statistically significant time points or turning points in the exposure-lag-response curve, to facilitate the identification of critical exposure windows^3,10^.

To evaluate the stability of the air pollution–PTB associations, we conducted sensitivity analyses by adjusting several key model parameters, including df and maximum lag time^3^. Additionally, to account for potential seasonal variation, we incorporated seasonal covariates into the main model.

R software version 4.3.1 was used to perform all statistical analyses, employing the “dlnm” and “mvmeta” packages.

## Results

### Descriptive analysis

**Fig. 2:**
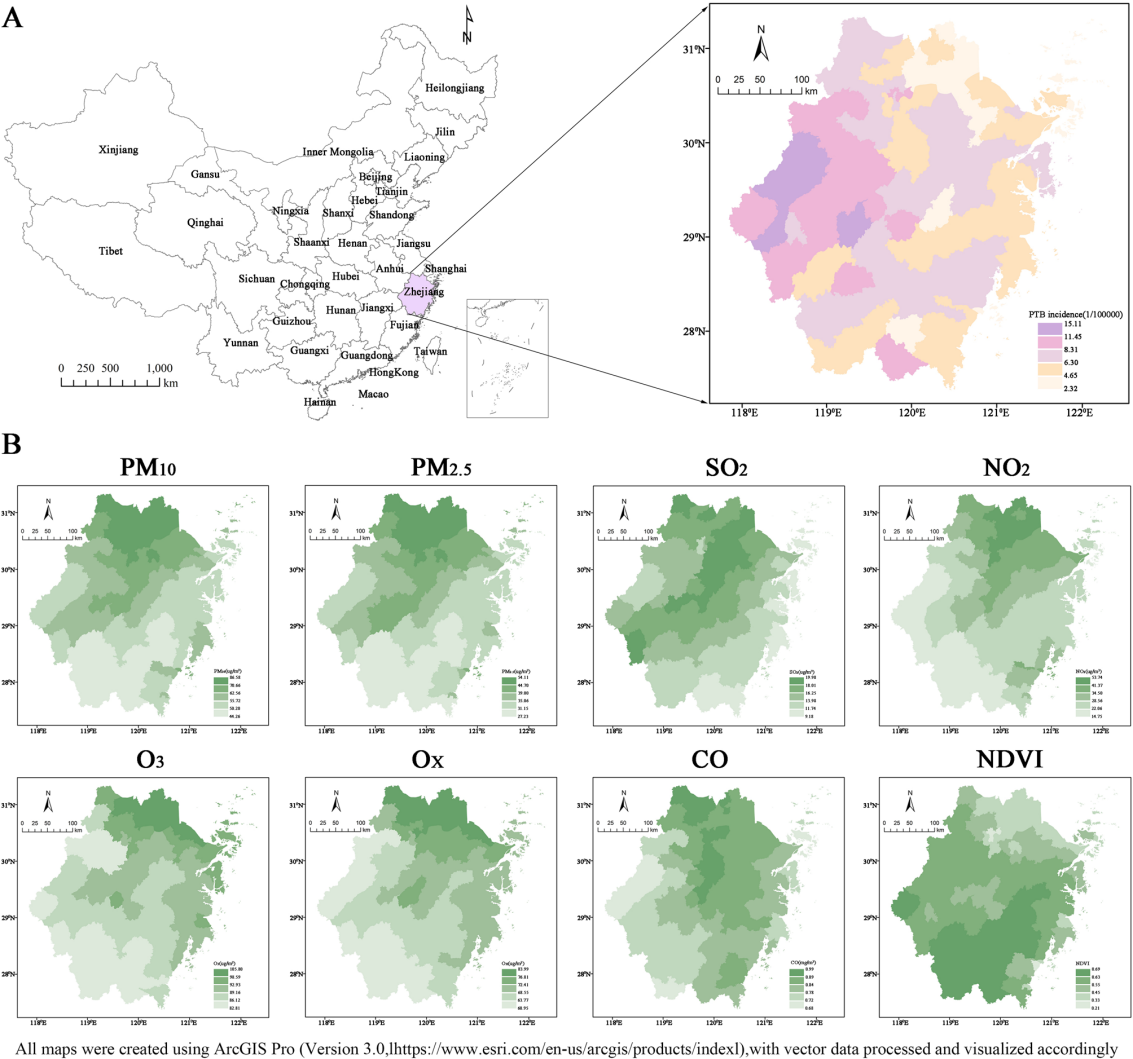
Spatial distribution of PTB incidence, average concentrations of eight air pollutants, and NDVI across 90 counties in Zhejiang Province from January 1, 2013, to December 31, 2019.

From January 2013 to December 2019, the average weekly PTB incidence was 0.685 per 100,000 persons. Weekly PTB incidence rates were 0.481 per 100,000 persons for males, 0.204 for females, 0.003 for children, 0.395 for individuals of working age, and 0.288 for the elderly (Table 1). These figures indicate a higher incidence of PTB among males and individuals in the working-age population. The average weekly concentrations of PM_10_, PM_2.5_, SO_2_, NO_2_, O_3_, O_X_, and CO were 62.08 µg/m^3^, 38.66 µg/ m^3^, 15.05 µg/m^3^, 31.39 µg/m^3^, 91.67 µg/m^3^, 71.16 µg/ m^3^, 0.83 mg/m^3^, respectively (Table 1). The incidence of PTB was higher in the western region of Zhejiang Province, China. Additionally, a spatial clustering pattern of increased air pollution levels was observed in the northwestern areas, while high NDVI values were concentrated in the southwest (Fig. 2). The cyclical pattern of the weekly distribution of air pollutants is evident from Fig. S1. Owing to its strong correlation with other variables and a significant decrease in Kappa values (from 235.6374 to 23.49235), PM_10_ was excluded from further analyses to mitigate issues of multicollinearity (Fig. S2).

**Table 1:** Overview of PTB incidence, ambient air pollutants, greenness, and meteorological factors in Zhejiang from 2013 to 2019.

Variables	Mean	SD	Centiles					
			Min	P25	P50	P75	Max	IQR
PTB Incidence (per 100,000)								
Total	0.685	0.515	0.000	0.318	0.606	0.943	7.105	0.625
Male	0.481	0.405	0.000	0.207	0.424	0.680	5.297	0.473
Female	0.204	0.242	0.000	0.000	0.159	0.311	4.522	0.311
Child, 0–14 (years)	0.003	0.026	0.000	0.000	0.000	0.000	1.107	0.000
Working-age population, 15–59 (years)	0.395	0.362	0.000	0.142	0.321	0.577	6.111	0.435
The elderly, ≥ 60 (years)	0.288	0.310	0.000	0.000	0.230	0.437	3.247	0.437
Ambient air pollutants								
PM_10_ (µg/m^3^)	62.08	27.07	17.07	42.92	56.48	74.99	337.51	32.07
PM_2.5_ (µg/m^3^)	38.66	19.20	8.36	25.49	34.49	47.31	260.31	21.83
SO_2_ (µg/m^3^)	15.05	8.97	3.32	8.81	12.59	18.56	211.75	9.75
NO_2_ (µg/m^3^)	31.39	14.14	5.78	20.78	28.76	39.47	119.95	18.69
O_3_ (µg/m^3^)	91.67	27.88	18.61	71.42	91.16	109.16	243.66	37.74
O_X_ (µg/m^3^)	71.16	17.62	25.30	58.55	69.64	81.76	171.96	23.21
CO (mg/m^3^)	0.83	0.20	0.34	0.69	0.80	0.93	3.82	0.24
Greenness								
NDVI	0.52	0.15	0.03	0.40	0.54	0.64	0.82	0.23
Meteorological factors								
precipitation (mm)	4.45	5.25	0.00	0.73	2.89	6.17	53.25	5.44
Temperature (◦C)	18.12	8.11	− 1.26	10.86	18.79	24.87	34.79	14.01
Wind speed (m/s)	2.16	0.73	0.40	1.68	2.06	2.51	7.46	0.82
Relative humidity (%)	76.44	9.13	38.39	70.50	77.20	83.22	98.52	12.72
Sunshine duration (h)	4.53	2.55	0.00	2.51	4.36	6.42	12.23	3.91

Min, minimum; Max, maximum; SD, standard deviation; IQR, interquartile range; P25, P50, and P75 represent the 25th, 50th, and 75th percentiles, respectively.

### Association between air pollutants and PTB incidence

#### Association between air pollutants and PTB incidence for total PTB cases

**Fig. 3:**
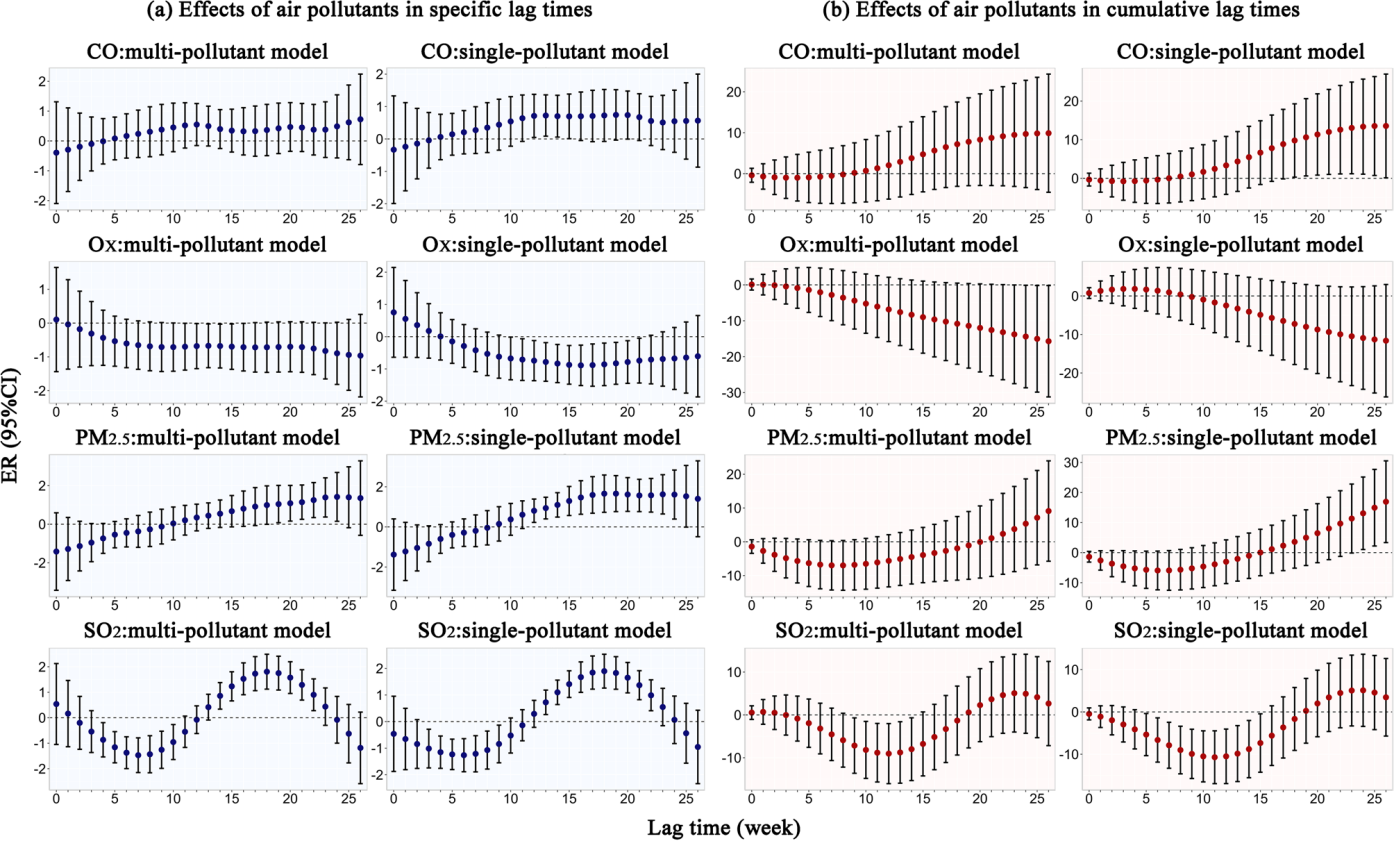
Effects of CO, O_X_, PM_2.5_ and SO_2_ on PTB risk at both specific and cumulative lag times.

Figure 3 illustrates the lag effects of each 10 µg/m^3^ increase in O_X_, PM_2.5_, and SO_2_, as well as each 0.1 mg/m^3^ increase in CO on PTB risk across specific and cumulative lag times from 0 to 26 weeks (Table S1 and Table S2). In the single-pollutant models, CO had a positive effect on PTB incidence. Specifically, for each 0.1 mg/m^3^ increase in CO, the PTB specific risk reached statistical significance with a 0.7% increase (95% CI 0.05%, 1.4%) at a 13-week lag, with cumulative ER increasing from 9.8% (95% CI 0.3%, 19.3%; lag 0–18) to 13.6% (95% CI 0.1%, 27.0%; lag 0–26). Conversely, O_X_ exhibited a negative association with PTB incidence, with a U-shaped lag specific risk curve and the lowest risk being a 0.9% decrease (95% CI − 1.5%, − 0.3%) for each 10 µg/m^3^ increase at a 16-week lag. Additionally, PM_2.5_ was positively correlated with PTB incidence. A 10 µg/m^3^ increase in PM_2.5_ was associated with an inverted U-shaped PTB specific risk curve, peaking at a 1.7% increase (95% CI 0.8%, 2.6%) at a 19-week lag. Furthermore, the cumulative ER of PTB incidence showed an increasing trend, ranging from 13.1% (95% CI 1.0%, 25.2%; lag 0–24) to 16.9% (95% CI 3.3%, 30.5%; lag 0–26). In contrast, SO_2_ had a negative effect on PTB incidence during weeks 3–9, but a positive effect during weeks 13–22 at specific lag times, forming an overall S-shaped risk curve. Additionally, for every 10 µg/m^3^ increase in SO_2_, the cumulative ER of PTB incidence showed an approximately U-shaped pattern within the statistically significant range, declining from − 5.4% (lag 0–5) to − 10.7% (lag 0–11), then rising to − 7.3% (lag 0–15). The lag effects of air pollutants on PTB incidence in the multi-pollutant model followed a comparable pattern to those observed in the single-pollutant model (Fig. 3).

#### Subgroup analysis

**Fig. 4:**
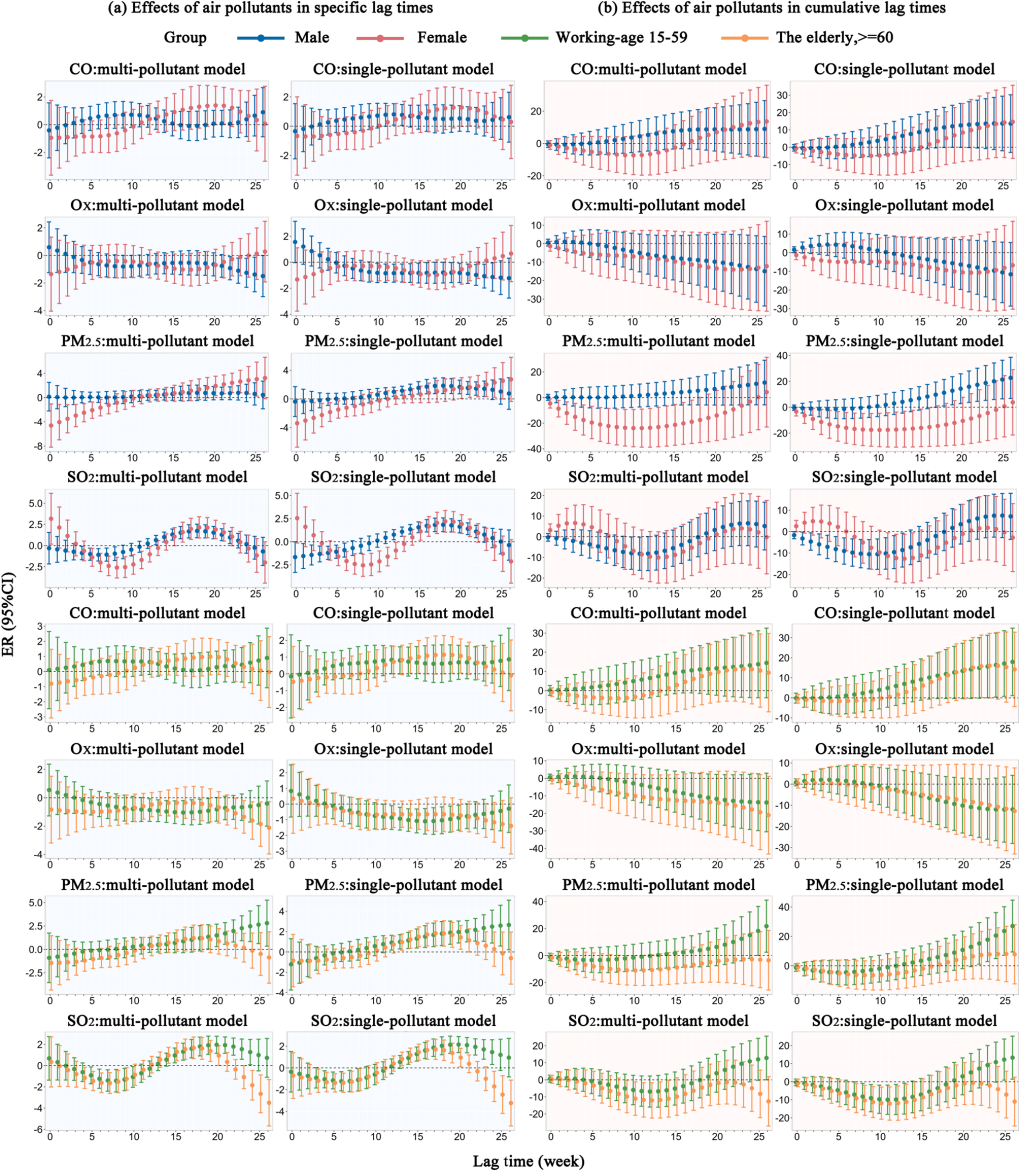
Effects of CO, O_X_, PM_2.5_, and SO_2_ on PTB risk at specific and cumulative lag times across various subgroups.

Figure 4 illustrates both the specific and cumulative risk of PTB associated with each 10 µg/m^3^ increase in O_X_, PM_2.5_, and SO_2_, as well as each 0.1 mg/m^3^ increase in CO across gender and age subgroups (Tables S3, S4, S5 and S6). CO was positively correlated with PTB incidence. Specifically, for each 0.1 mg/m^3^ increase in CO, PTB specific risk increased significantly at a single lag point: by 0.8% (95% CI 0.1%, 1.5%; lag 12) in males, by 1.2% (95% CI 0.01%, 2.4%; lag 21) in females, by 0.8% (95% CI 0.01%, 1.7%; lag 12) in working-age adults, and by 0.9% (95% CI 0.04%, 1.8%; lag 14) in the elderly, respectively. The cumulative ER of PTB incidence showed an upward trend, increasing from 15.1% (lag 0–21) to 15.7% (lag 0–22) in the elderly subgroup, and from 15.7% (lag 0–23) to 18.0% (lag 0–26) in the working-age subgroup.

O_X_ was negatively correlated with PTB incidence in the male and working-age subgroups. For every 10 µg/m^3^ increase in O_X_, the PTB specific ER exhibited a U-shaped pattern within statistically significant results, with the lowest risk observed as a 0.9% decrease (95% CI − 1.7%, − 0.05%; lag 17) in the male subgroup and a 1.1% decrease (95% CI − 1.9%, − 0.3%; lag 16) in the working-age subgroup.

For each 10 µg/m^3^ increase in PM_2.5_, the lag-specific ER of PTB incidence exhibited an inverted U-shaped pattern within lag periods showing statistically significant associations among males and the elderly, whereas an increasing trend was observed among females and working-age adults. The peak ERs were 1.8% (95% CI 0.7%, 2.9%; lag 18) in males, 1.7% (95% CI 0.5%, 2.9%; lag 19) in the elderly, 2.5% (95% CI 0.01%, 5.1%; lag 25) in females, and 2.7% (95% CI 0.2%, 5.1%; lag 25) in working-age adults. The cumulative ER of PTB incidence exhibited an increase from 15.2% (lag 0–22) to 27.0% (lag 0–26) in the working-age subgroup and from 11.2% (lag 0–18) to 22.7% (lag 0–26) in the male subgroup. Conversely, in the female subgroup, the cumulative ER of PTB incidence followed a U-shaped pattern, reaching a minimum of − 10.6% (lag 0–10).

For each 10 µg/m^3^ increase in SO_2_, different associations with PTB incidence were observed across subgroups during specific lag times. In the male subgroup, the specific risk curve showed an overall inverted U-shape, with a negative association between SO_2_ and PTB incidence during weeks 0–6 and a positive association during weeks 12–22. In contrast, the female, working-age, and elderly subgroups exhibited S-shaped risk curves, characterized by negative associations during weeks 5–12, 4–8, and 3–8, respectively, followed by positive associations during weeks 15–21, 14–24, and 13–20. Additionally, in all subgroups, the cumulative ER of PTB incidence showed a U-shaped trend across lag periods with statistically significant associations. The lowest cumulative ERs were observed at lag 9 in males (− 10.5%), lag 13 in females (− 12.6%), lag 11 in working-age adults (− 10.0%), and lag 11 in the elderly (− 12.1%).

### Modification effects of NDVI

**Fig. 5:**
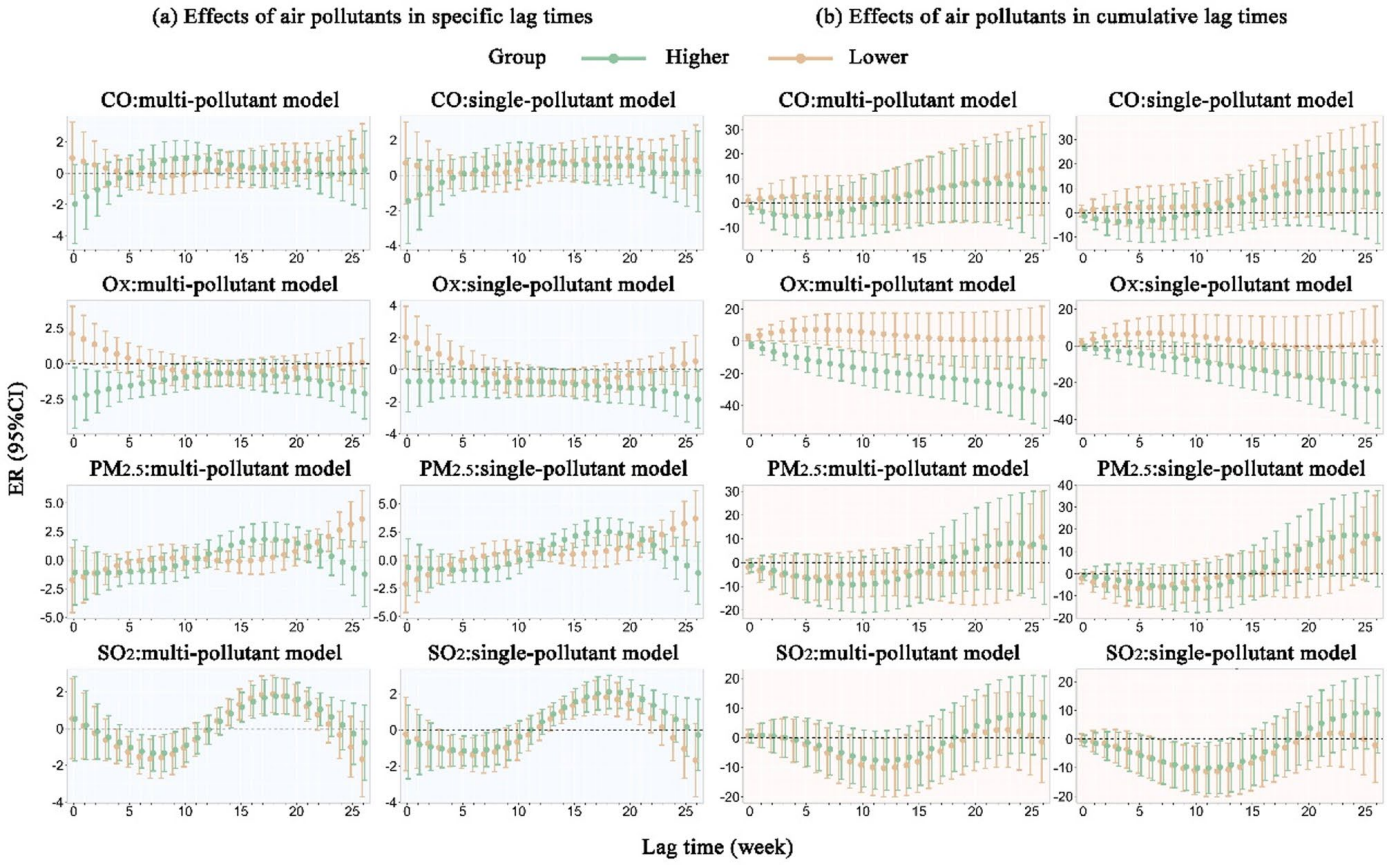
Effects of CO, O_X_, PM_2.5_, and SO_2_ on PTB risk at specific and cumulative lag times across different NDVI levels.

Figure 5 illustrates both the specific and cumulative PTB risk associated with a 10 µg/m^3^ increase in O_X_, PM_2.5_, and SO_2_, as well as a 0.1 mg/m^3^ increase in CO, in areas with lower and higher NDVI values (Tables S7 and S8). In the single-pollutant model, CO showed a positive effect with PTB incidence in areas with lower NDVI values. For each 0.1 mg/m^3^ increase in CO, PTB specific ER increased significantly by 1% (95% CI 0.03%, 2.0%) only at a 22-week lag, while the cumulative ER of PTB incidence showed an increasing trend, rising from 17.9% (lag 0–24) to 19.3% (lag 0–26).

O_X_ was positively correlated with PTB incidence in areas with lower NDVI values. For each 10 µg/m^3^ increase in O_X_, PTB specific risk increased by 2.0% (95% CI 0.1%, 3.9%) only at lag 0 week, while the cumulative ER of PTB incidence increasing from 2.0% (lag 0) to 3.7% (lag 0–1). Conversely, in areas with higher NDVI values, O_X_ was negatively correlated with PTB incidence. A statistically significant 0.8% decrease in PTB risk (95% CI − 1.7%, − 0.01%) was observed at lag 14, with cumulative ER decreasing from − 10.8% (lag 0–13) to − 24.7% (lag 0–26).

PM_2.5_ was positively correlated with PTB risk in both lower- and higher-NDVI areas. For every 10 µg/m^3^ increase in PM_2.5_, PTB specific risk showed an upward trend, peaking at a 3.7% increase (95% CI 1.2%, 6.2%) at a lag of 26 weeks, while the cumulative ER of PTB incidence increased by 17.7% (lag 0–26) in lower-NDVI areas. In higher-NDVI areas, PTB specific risk exhibited an inverted U-shaped pattern across lag periods with statistically significant associations, peaking at a 2.5% increase (95% CI 1.3%, 3.7%) at a lag of 17 weeks.

For every 10 µg/m^3^ increase in SO_2_, the PTB-specific risk curves showed an overall S-shaped pattern in both low- and high-NDVI areas. In low-NDVI areas, SO₂ exposure was negatively associated with PTB incidence during lags 4–8 and positively associated during lags 14–21. In high-NDVI areas, similar associations were observed during lags 3–7 and 13–23, respectively. The cumulative ER exhibited a U-shaped trend, with the lowest risks being − 11.3% at lag 11 (low NDVI) and − 10.1% at lag 10 (high NDVI).

### Sensitivity analysis

In the sensitivity analyses (Figs. S3, S4, and S5), the inclusion of season covariates, modifying the degrees of freedom for meteorological factors, or adjusting the lag period to a maximum of 20 or 30 weeks did not significantly affect the main findings. These results indicated that our study achieved robust model fitting outcomes.

## Discussion

This study was a time-series investigation that examined both short- and long-term lagged effects of multiple air pollutants on PTB incidence, as well as the modifying effects of greenness over different lag periods. We found that SO_2_ has a dual association with PTB incidence, encompassing both a short-term negative correlation and a long-term positive correlation. PTB incidence exhibited a positive correlation with long-term exposure to CO and PM_2.5_, while demonstrating a negative association with long-term O_X_ exposure. The correlations between CO and PM_2.5_ and PTB incidence were more pronounced in the male and working-age subgroups, while associations with SO_2_ were stronger in the female and elderly subgroups. The stratified analyses revealed that greenness modified the associations between SO_2_ and O_X_ exposure and PTB incidence across various lag times.

Both a short-term negative association and a long-term positive association between SO_2_ exposure and PTB incidence were observed. On one hand, a study conducted in Anhui Province^19, ^China, also observed a negative correlation between short-term SO_2_ exposure and TB incidence. SO_2_ oxidation capability rapidly kills MTB by inducing oxidative damage to bacterial molecular structures, such as lipids, proteins, and DNA^42,43^. Moreover, the acute protective effects of short-term SO_2_ exposure on PTB incidence can be attributed to its antibacterial properties, which likely stem from its ability to disrupt enzyme activities within the cell membranes of microorganisms, thereby swiftly affecting the physiological functions of MTB^25^. However, several studies conducted in Wulumuqi City^14^ and Lianyungang City^12, ^China, have demonstrated a positive correlation between long-term exposure to SO_2_ and TB incidence. Owing to its high solubility in water, SO_2_ can damage the mucosa of the upper respiratory tract^44^. In the early stages of infection, alveolar macrophages are crucial for inhibiting the proliferation of MTB through phagocytosis and granuloma formation within the pulmonary system^44^. However, SO_2_ exposure can impair the function of macrophage, thereby affecting alveolar clearance and ciliary transport^45^. Moreover, long-term SO_2_ exposure may reduce tumor necrosis factor (TNF)-α^46^which is essential for combating MTB infection^47^.

For every 10 µg/m^3^ increase in O_X_, the lowest PTB risk was observed as a 0.9% decrease (95% CI − 1.5%, − 0.3%) at lag week 16. The interchange between NO_2_ and O_3_ as potent oxidants over short timescales supports their combination into the pollutant O_X_, simplifying health effect assessments while capturing their simultaneous effects^48^. Several studies conducted in Hefei^18^ and Beijing^3, ^China, have identified a negative association between O_3_ exposure and TB incidence. Additionally, a quasi-experimental study has shown that O_3_ can improve cellular redox balance and enhance oxygen absorption capacity, thereby strengthening human immune function^49^. Furthermore, previous studies have reported a detrimental effect of NO_2_ on TB incidence^50,51^. NO_2_ can induce damage to the respiratory mucosa and impair ciliary clearance function, which can induce respiratory symptoms and compromising the immune system^52–54^. Our findings further indicate that O_3_ exerts a stronger protective effect when considering the combined effect of NO_2_ and O_3_. Additionally, we found that long-term exposure to PM_2.5_ and CO is associated with increased PTB incidence, a relationship supported by previous research^55,56^.

The subgroup analysis revealed that males and working-age groups are more susceptible to the harmful effects of air pollution on PTB incidence. This increased vulnerability is likely due to their larger vital capacity and higher exertion rates from engaging in labor-intensive occupations, compared with females and elderly individuals. These factors are closely related to a greater susceptibility to ambient air pollution^57^. Additionally, males are more likely to encounter adverse lifestyle factors such as smoking and excessive alcohol consumption, which can contribute to the development of active TB in this subgroup^58^.

Our study observed that greenness modified the association between O_X_ and PTB incidence at various lag times. Previous studies have identified the influence of greenness on the associations between long-term air pollution exposure and TB risk^28,29^. However, there is limited research on how greenness modifies the influence of short-term air pollution exposure on PTB incidence. Our findings revealed that in areas with lower NDVI, a positive correlation between short-term O_X_ exposure and PTB incidence was observed, whereas in areas with higher NDVI, there was a negative association between long-term exposure to O_X_ and PTB incidence. Several potential mechanisms could explain this phenomenon. First, numerous studies have demonstrated the acute detrimental effect of NO_2_ exposure on TB incidence^10,18,59, ^likely due to its role as a major contributor to photochemical smog and its acute pulmonary toxicity^30^. Additionally, research suggests that greenness can help mitigate NO_2_ concentrations through wet and dry deposition, alteration of airflow patterns, and acceleration of chemical transformations^60,61^. In areas with low NDVI, the reduced vegetation reduces the effectiveness of these mitigation processes for NO_2_, which mainly originates from fuel combustion, agricultural activities, and traffic emissions^62, ^leading to increased NO_2_ concentrations. Consequently, the harmful effects of NO_2_ prevail over those of O_3_ in areas with low NDVI. Second, time-series analyses have confirmed an inverse relationship between O_3_ concentration and TB incidence^63^. This association is likely due to O_3_ ability to enhance cellular redox balance and oxygen uptake^49^. Additionally, vegetation can influence O_3_ levels by reacting with biogenic volatile organic compounds emitted by plants and nitrogen oxides^64^. Additionally, the average O_3_ concentration in Zhejiang Province during the study period was relatively low, at 91.67 µg/m^3^. As a result, in areas with higher NDVI, the protective effect of O_3_ predominates over the combined effects of NO_2_ and O_3_, mainly due to the increase in O_3_ concentration even at relatively low levels.

By simultaneously analyzing both short- and long-term lag effects, our study offers a more precise quantification of the risk that air pollution poses to PTB incidence, thereby minimizing potential biases that could arise from considering only a single time dimension. Moreover, greenness was identified as a modifier in the association between short-term air pollution exposure and PTB incidence, addressing a significant gap in the existing literature on TB risk. Additionally, our research aims to elucidate the potential mechanisms through which air pollutants affect PTB incidence in Zhejiang Province, providing valuable insights for the prevention and control of PTB in developing countries.

In this study, several potential limitations should be acknowledged. First, the effect of ambient air pollutants on PTB incidence is influenced by individual characteristics such as lifestyle and health status^65^. However, due to the ecological design of the study, individual-level differences in exposure, health conditions, and covariate data could not be accounted for. Moreover, assigning pollution levels at the county level may introduce misclassification, as the county level may fail to capture within-county variation, especially in geographically large or environmentally heterogeneous areas. This limitation reflects a broader issue known as the Modifiable Areal Unit Problem (MAUP), whereby the choice of spatial aggregation units can influence statistical associations. Future research may benefit from using smaller scale exposure data or spatial analysis models to address this challenge. Second, this study did not account for the infectiousness of PTB, which may influence incidence patterns. Although we included seasonal indicators to partially control for seasonal variation, these adjustments may not fully capture transmission dynamics. Future research should consider integrating environmental exposure assessments with infectious disease transmission models to more comprehensively capture the interplay between environmental risk factors and PTB transmission dynamics. Third, meteorological data from monitoring stations were interpolated using the inverse distance weighting (IDW) method, which could introduce discrepancies due to factors such as topography, vegetation, and human activities. However, since these data primarily serve as covariates, their influence on the overall results is likely minimal. Fourth, using weekly averages instead of daily averages may overlook the potential effects of extreme weather events or fluctuations in air pollution on TB incidence. Finally, the modification effect of greenness may vary depending on its coverage, types, and characteristics^64^. For example, urban parks and suburban forests are more effective in reducing SO_2_ and NO_X_ emissions^66,67^. However, using NDVI alone provides only a broad estimation of greenness coverage. To improve future research, it is essential to evaluate different types and characteristics of greenness. This approach would allow for a more detailed examination of potential modifications and offer more specific guidance for greenspace planning.

## Conclusions

In this time-series analysis, we identified a dual association between SO_2_ exposure and PTB incidence, with a short-term negative association and a long-term positive association. We also observed positive correlations between long-term exposure to CO and PM_2.5_ and PTB incidence, while long-term O_X_ exposure was negatively associated with PTB incidence. These findings underscore the need for government policies that focus on long-term control strategies, including enhanced monitoring and emission management of PM_2.5_ and CO. Additionally, establishing a robust environmental monitoring system with an early warning mechanism is crucial to address the lagged effects of both short- and long-term SO_2_ exposure. Furthermore, our findings show that exposure to greenness has beneficial effects on county-level PTB incidence. These insights are valuable for guiding green infrastructure development, garden afforestation, and policy-making aimed at reducing the burden of PTB.

## Electronic supplementary material

Below is the link to the electronic supplementary material.

Supplementary Material 1

## Data Availability

All data generated or analysed are included in this article and its supplementary information files. The corresponding authors can provide data upon reasonable request after completing all studies and sub-studies.

## References

[1] Li, Z. Q. et al. Meteorological factors contribute to the risk of pulmonary tuberculosis: A multicenter study in Eastern China. *Sci. Total Environ.* **793**, 8 (2021). 10.1016/j.scitotenv.2021.148621 34328976

[2] Wu, D. W. et al. Impact of the synergistic effect of pneumonia and air pollutants on newly diagnosed pulmonary tuberculosis in Southern Taiwan. *Environ. Res.* **212**, 11 (2022). 10.1016/j.envres.2022.113215 35367429

[3] Sun, S. H. et al. The association between air pollutants, meteorological factors and tuberculosis cases in beijing, china: A seven-year time series study. *Environ. Res.* **216**, 9 (2023). 10.1016/j.envres.2022.114581 36244443

[4] WHO. *Global Tuberculosis Report 2023* (World Health Organization, 2023).

[5] Smith, G. S., Schoenbach, V. J., Richardson, D. B. & Gammon, M. D. Particulate air pollution and susceptibility to the development of pulmonary tuberculosis disease in North carolina: an ecological study. *Int. J. Environ. Health Res.* **24**, 103–112 (2014). 24387197 10.1080/09603123.2013.800959 PMC4364606

[6] Yi, X. & Liu, S. X. Impact of environmental factors on pulmonary tuberculosis in multi-levels industrial upgrading area of China. *Environ. Res.* **195**, 12 (2021). 10.1016/j.envres.2021.110768 33548291

[7] Cao, F. et al. Effects of meteorological factors and air pollutants on the incidence of pulmonary tuberculosis in Yulin from 2017 to 2021. *Chin. J. Antituberculosis*. **44**, 1154–1161 (2022).

[8] Crouse, D. L. et al. Complex relationships between greenness, air pollution, and mortality in a population-based Canadian cohort. *Environ. Int.* **128**, 292–300 (2019). 31075749 10.1016/j.envint.2019.04.047

[9] Kharwadkar, S., Attanayake, V., Duncan, J., Navaratne, N. & Benson, J. The impact of climate change on the risk factors for tuberculosis: A systematic review. *Environ. Res.* **212**, 12 (2022). 10.1016/j.envres.2022.113436 35550808

[10] Feng, Y. Q. et al. Lagged effects of exposure to air pollutants on the risk of pulmonary tuberculosis in a highly polluted region. *Int. J. Environ. Res. Public. Health*. **19**, 13 (2022). 10.3390/ijerph19095752 PMC9106023 35565147

[11] Deng, X. Y. et al. The short-term effect of air pollution on the incidence of pulmonary tuberculosis in chongqing, china, 2014–2020. *J. Infect. Dev. Ctries.* **17**, 1722– (2023). 38252717 10.3855/jidc.17217

[12] Li, Z. Q. et al. Long-term effect of exposure to ambient air pollution on the risk of active tuberculosis. *Int. J. Infect. Dis.* **87**, 177–184 (2019). 31374344 10.1016/j.ijid.2019.07.027

[13] Wang, X. Q. et al. Short-term effect of ambient air pollutant change on the risk of tuberculosis outpatient visits: a time-series study in fuyang, China. *Environ. Sci. Pollut Res.* **29**, 30656–30672 (2022). 10.1007/s11356-021-17323-7 34993790

[14] Yang, J. D. et al. A study on the relationship between air pollution and pulmonary tuberculosis based on the general additive model in wulumuqi, China. *Int. J. Infect. Dis.* **96**, 42–47 (2020). 32200108 10.1016/j.ijid.2020.03.032

[15] Li, Z. Q. et al. Ambient air pollution contributed to pulmonary tuberculosis in China. *Emerg. Microbes Infect.* **13**, 9 (2024). 10.1080/22221751.2024.2399275 PMC11378674 39206812

[16] Liu, Y. et al. Ambient air pollution exposures and risk of drug-resistant tuberculosis. *Environ. Int.* **124**, 161–169 (2019). 30641260 10.1016/j.envint.2019.01.013

[17] Alvaro-Meca, A., Díaz, A., Díez, J. D. M., Resino, R. & Resino, S. Environmental factors related to pulmonary tuberculosis in HIV-Infected patients in the combined antiretroviral therapy (cART) era. *PLoS One*. **11**, 14 (2016). 10.1371/journal.pone.0165944 PMC5094733 27812194

[18] Huang, K. et al. Association between short-term exposure to ambient air pollutants and the risk of tuberculosis outpatient visits: A time-series study in hefei, China. *Environ. Res.* **184**, 9 (2020). 10.1016/j.envres.2020.109343 32192989

[19] Wang, X. Q. et al., Short-term effect of sulfur dioxide (SO_2_) change on the risk of tuberculosis outpatient visits in 16 cities of Anhui Province, China: the first multi-city study to explore differences in occupational patients. *Environ. Sci. Pollut. Res*. **13** (2022). 10.1007/s11356-022-19438-x PMC8882443 35224697

[20] Nie, Y. W. et al. Interaction between air pollutants and meteorological factors on pulmonary tuberculosis in Northwest china: A case study of eight districts in Urumqi. *Int. J. Biometeorol.* **68**, 691–700 (2024). 38182774 10.1007/s00484-023-02615-z

[21] Liu, F. Q., Zhang, Z. X., Chen, H. Y. & Nie, S. F. Associations of ambient air pollutants with regional pulmonary tuberculosis incidence in the central Chinese Province of hubei: a bayesian spatial-temporal analysis. *Environ. Health*. **19**, 10 (2020). 32410699 10.1186/s12940-020-00604-y PMC7226955

[22] Tao, B. L. et al. Environment pollutants exposure affects the endogenous activation of within-host Mycobacterium tuberculosis. *Environ. Res.* **227**, 10 (2023). 10.1016/j.envres.2023.115695 36958381

[23] Lin, Y. J. et al. Association between ambient air pollution and elevated risk of tuberculosis development. *Infect. Drug Resist.* **12**, 3835–3847 (2019). 31827330 10.2147/IDR.S227823 PMC6902850

[24] Niu, Z. C. et al. Short-term effects of ambient air pollution and meteorological factors on tuberculosis in semi-arid area, Northwest china: a case study in Lanzhou. *Environ. Sci. Pollut. Res.* **28**, 69190–69199 (2021). 10.1007/s11356-021-15445-6 34291414

[25] Popovic, I. et al. A systematic literature review and critical appraisal of epidemiological studies on outdoor air pollution and tuberculosis outcomes. *Environ. Res.* **170**, 33–45 (2019). 30557690 10.1016/j.envres.2018.12.011

[26] Markevych, I. et al. Exploring pathways linking greenspace to health: theoretical and methodological guidance. *Environ. Res.* **158**, 301–317 (2017). 28672128 10.1016/j.envres.2017.06.028

[27] Guo, T. L. et al. Association of fine particulate matter and residential greenness with risk of pulmonary tuberculosis retreatment: population-based retrospective study. *JMIR Public. Health Surveill*. **10**, 15 (2024). 10.2196/50244 PMC11337066 39140280

[28] Ge, E. J. et al. Effect modification of greenness on PM_2.5_ associated all-cause mortality in a multidrug-resistant tuberculosis cohort. *Thorax*. **77**, 1202–1209 (2022). 34876501 10.1136/thoraxjnl-2020-216819

[29] Zhu, S. et al. Long-term exposure to ambient air pollution and greenness in relation to pulmonary tuberculosis in china: A nationwide modelling study. *Environ. Res.* **214**, 10 (2022). 10.1016/j.envres.2022.114100 35985487

[30] Wang, X. M., Chen, J. M., Cheng, T. T., Zhang, R. Y. & Wang, X. M. Particle number concentration, size distribution and chemical composition during haze and photochemical smog episodes in Shanghai. *J. Environ. Sci.* **26**, 1894–1902 (2014). 10.1016/j.jes.2014.07.003 25193840

[31] Zhao, J. W. et al. Effect of gaseous pollutant and greenness exposure on mortality during treatment of newly treated tuberculosis patients: a provincial population-based cohort study. *Environ. Sci. Pollut. Res.* **30**, 98195–98210 (2023). 10.1007/s11356-023-29256-4 37608175

[32] Wang, W. J. et al. Epidemiological characteristics of tuberculosis and effects of meteorological factors and air pollutants on tuberculosis in shijiazhuang, china: A distribution lag non-linear analysis. *Environ. Res.* **195**, 11 (2021). 10.1016/j.envres.2020.110310 33098820

[33] Zhu, S. et al. Ambient air pollutants are associated with newly diagnosed tuberculosis: A time-series study in chengdu, China. *Sci. Total Environ.* **631–632**, 47–55 (2018). 29524902 10.1016/j.scitotenv.2018.03.017

[34] Bratsch, S. G. Standard electrode-potentials and temperature coefficients in water at 298.15-K. *J. Phys. Chem. Ref. Data*. **18**, 1–21 (1989).

[35] Wang, X. Q. et al. Associations of exposures to air pollution and greenness with mortality in a newly treated tuberculosis cohort. *Environ. Sci. Pollut. Res.* **30**, 34229–34242 (2023). 10.1007/s11356-022-24433-3 PMC9742034 36504301

[36] Konopka, K. et al. Combined neutrophil-to-lymphocyte and platelet-volume-to-platelet ratio (NLR and PVPR Score) represents a novel prognostic factor in advanced gastric Cancer patients. *J. Clin. Med.* **10**, 11 (2021). 10.3390/jcm10173902 PMC8432226 34501353

[37] Huang, K. et al. Contributions of ambient temperature and relative humidity to the risk of tuberculosis admissions: A multicity study in central China. *Sci. Total Environ.* **838**, 9 (2022). 10.1016/j.scitotenv.2022.156272 35644395

[38] Liu, Y. et al. Effect of ambient air pollution on tuberculosis risks and mortality in shandong, china: a multi-city modeling study of the short- and long-term effects of pollutants. *Environ. Sci. Pollut. Res.* **28**, 27757–27768 (2021). 10.1007/s11356-021-12621-6 33515408

[39] Mohidem, N. A. et al. Association of sociodemographic and environmental factors with Spatial distribution of tuberculosis cases in gombak, selangor, Malaysia. *PLoS One*. **16**, 32 (2021). 10.1371/journal.pone.0252146 PMC8211220 34138899

[40] Xie, J. G. & Zhu, Y. J. Association between ambient temperature and COVID-19 infection in 122 cities from China. *Sci. Total Environ.* **724**, 5 (2020). 10.1016/j.scitotenv.2020.138201 PMC7142675 32408450

[41] Zhu, Y. J., Xie, J. G., Huang, F. M. & Cao, L. Q. Association between short-term exposure to air pollution and COVID-19 infection: evidence from China. *Sci. Total Environ.* **727**, 7 (2020). 10.1016/j.scitotenv.2020.138704 PMC7159846 32315904

[42] Malwal, S. R. et al. Synthesis, and evaluation of Thiol-Activated sources of sulfur dioxide (SO_2_) as antimycobacterial agents. *J. Med. Chem.* **55**, 553–557 (2012). 22128803 10.1021/jm201023g

[43] Rencüzogullari, E., Ila, H. B., Kayraldiz, A. & Topaktas, M. Chromosome aberrations and sister chromatid exchanges in cultured human lymphocytes treated with sodium metabisulfite, a food preservative. *Mutat. Res. Genet. Toxicol. Environ. Mutagen.* **490**, 107–112 (2001). 10.1016/s1383-5718(00)00142-x 11342236

[44] Palanisamy, G. S. et al. Evidence for oxidative stress and defective antioxidant response in Guinea pigs with tuberculosis. *PLoS One*. **6**, 13 (2011). 10.1371/journal.pone.0026254 PMC3196542 22028843

[45] Hwang, S. S. et al. Impact of outdoor air pollution on the incidence of tuberculosis in the Seoul metropolitan area, South Korea. *Korean J. Intern. Med.* **29**, 183–190 (2014). 24648801 10.3904/kjim.2014.29.2.183 PMC3956988

[46] Saito, Y., Azuma, A., Kudo, S., Takizawa, H. & Sugawara, I. Effects of diesel exhaust on murine alveolar macrophages and a macrophage cell line. *Exp. Lung Res.* **28**, 201–217 (2002). 11936774 10.1080/019021402753570509

[47] Dutta, N. K. & Karakousis, P. C. Latent tuberculosis infection: myths, models, and molecular mechanisms. *Microbiol. Mol. Biol. Rev.* **78**, 343–371 (2014). 25184558 10.1128/MMBR.00010-14 PMC4187682

[48] Williams, M. L., Atkinson, R. W., Anderson, H. R. & Kelly, F. J. Associations between daily mortality in London and combined oxidant capacity, Ozone and nitrogen dioxide. *Air Qual. Atmos. Health*. **7**, 407–414 (2014). 25431629 10.1007/s11869-014-0249-8 PMC4239710

[49] Shah, M. A., Anande, L. K., Powar, A. & Captain, J. & Mk nair, P. The role of medical Ozone in improving antioxidant status in multiple Drug-Resistant tuberculosis patients: A Quasi-experimental study. **6**, e97125 (2019).

[50] Xiang, K. et al. Association between ambient air pollution and tuberculosis risk: A systematic review and meta-analysis. *Chemosphere*. **277**, 12 (2021). 10.1016/j.chemosphere.2021.130342 33794431

[51] Giri, N. et al. Disease Migration, Mitigation, and Containment: Impact of Climatic Conditions & Air Quality on Tuberculosis for India. In *2nd IEEE-Pune-Section Annual International Conference (IEEE PuneCon) (Ieee, MIT World Peace Univ, Sch Elect & Commun, MIT Coll Engn, Pune, India* (2019).

[52] Xu, M. et al. Association of air pollution with the risk of initial outpatient visits for tuberculosis in wuhan, China. *Occup. Environ. Med.* **76**, 560–566 (2019). 31300562 10.1136/oemed-2018-105532

[53] Glencross, D. A., Ho, T. R., Camilla, N., Hawrylowicz, C. M. & Pfeffer, P. E. Air pollution and its effects on the immune system. *Free Radic. Biol. Med.* **151**, 56–68 (2020). 32007522 10.1016/j.freeradbiomed.2020.01.179

[54] D’Amato, G., Cecchi, L., D’Amato, M. & Liccardi, G. Urban air pollution and climate change as environmental risk factors of respiratory allergy: an update. *J. Investig. Allergol. Clin. Immunol.* **20**, 95–102 (2010). 20461963

[55] Xu, M. et al. Association of long-term exposure to ambient air pollution with the number of tuberculosis cases notified: a time-series study in Hong Kong. *Environ. Sci. Pollut Res.* **29**, 21621–21633 (2022). 10.1007/s11356-021-17082-5 34767173

[56] Chen, N., Lindsey, G. & Wang, C. H. Patterns and correlates of urban trail use: evidence from the Cincinnati metropolitan area. *Transp. Res. Part. D: Transp. Environ.* **67**, 303–315 (2019).

[57] Ge, E. J. et al. Ambient sulfur dioxide levels associated with reduced risk of initial outpatient visits for tuberculosis: A population based time series analysis. *Environ. Pollut*. **228**, 408–415 (2017). 28554030 10.1016/j.envpol.2017.05.051

[58] Przybylski, G., Nowakowska-Arendt, A., Pilaczynska-Cemel, M. & Golda, R. 10 years comparative clinico-epidemiological analysis of smoking and alcohol consumption in TB patients (Myc. Tuberculosis) and with mycobacteriosis (Myc. Kansas. *Przegl. Lek.* **71**, 576–580 (2014). 25799847

[59] Zhao, C. N. et al. Associations between air pollutants and acute exacerbation of drug-resistant tuberculosis: evidence from a prospective cohort study. *BMC Infect. Dis.* **24**, 9 (2024). 38262983 10.1186/s12879-024-09011-x PMC10807089

[60] Rao, M., George, L. A., Rosenstiel, T. N., Shandas, V. & Dinno, A. Assessing the relationship among urban trees, nitrogen dioxide, and respiratory health. *Environ. Pollut*. **194**, 96–104 (2014). 25103043 10.1016/j.envpol.2014.07.011

[61] Cai, L. Y., Zhuang, M. Z. & Ren, Y. Spatiotemporal characteristics of NO_2_, PM_2.5_ and O_3_ in a coastal region of southeastern China and their removal by green spaces. *Int. J. Environ. Health Res.* **32**, 1–17 (2022). 32013546 10.1080/09603123.2020.1720620

[62] Chen, T. H. et al. A hybrid kriging/land-use regression model with Asian culture-specific sources to assess NO_2_ spatial-temporal variations. *Environ. Pollut*. **259**, 10 (2020). 10.1016/j.envpol.2019.113875 31918142

[63] Wang, H., Tian, C., Wang, W. & Luo, X. Temporal Cross-Correlations between ambient air pollutants and seasonality of tuberculosis: A time-series analysis. **16**, 1585 (2019). 10.3390/ijerph16091585 PMC6540206 31064146

[64] Gong, C., Xian, C. F., Wu, T., Liu, J. R. & Ouyang, Z. Y. Role of urban vegetation in air phytoremediation: differences between scientific research and environmental management perspectives. *NPJ Urban Sustain.* **3**, 15 (2023).36936645

[65] Bates, M. N. et al. Household fuel use and pulmonary tuberculosis in Western nepal: A case-control study. *Environ. Res.* **168**, 193–205 (2019). 30317104 10.1016/j.envres.2018.09.036 PMC6263816

[66] Gong, C. et al. Estimating NOx removal capacity of urban trees using stable isotope method: A case study of beijing, China. *Environ. Pollut*. **290**, 11 (2021). 10.1016/j.envpol.2021.118004 34454196

[67] Douglas, A. N. J., Irga, P. J. & Torpy, F. R. Determining broad scale associations between air pollutants and urban forestry: A novel multifaceted methodological approach. *Environ. Pollut*. **247**, 474–481 (2019). 30690244 10.1016/j.envpol.2018.12.099

