# Supplementary material for: Short- and long-term exposure to ambient air pollution and greenness in relation to pulmonary tuberculosis incidence
Source: Sci Rep. 2025 Jul 15;15:25594. doi: 10.1038/s41598-025-11465-1 (PMC12264143; doi:10.1038/s41598-025-11465-1)
Supplement: Supplementary file 1 — Supplementary Material 1 [file 41598_2025_11465_MOESM1_ESM.pdf]

Supplementary tables and figures

Contents

Table S1. The average scores of Quasi-Akaike Information Criterion for the maximum lag period in 90 counties of Zhejiang Province, China, from 2013 to 2019.

Table S2. Changes in the excess risk of pulmonary tuberculosis incidence and their 95% CIs for every 10 μg/m^3 increase in O_X, $PM_{2.5}$ and SO_2, and every 0.1 mg/m^3 increase in CO in Zhejiang Province, China, from 2013 to 2019.

Table S3. Changes in the excess risk of pulmonary tuberculosis incidence and their 95% CIs for every 10 μg/m^3 increase in O_X, $PM_{2.5}$ and SO_2, and every 0.1 mg/m^3 increase in CO in male subgroup.

Table S4. Changes in the excess risk of pulmonary tuberculosis incidence and their 95% CIs for every 10 μg/m^3 increase in O_X, $PM_{2.5}$ and SO_2, and every 0.1 mg/m^3 increase in CO in female subgroup.

Table S5. Changes in the excess risk of pulmonary tuberculosis incidence and their 95% CIs for every 10 μg/m^3 increase in O_X, $PM_{2.5}$ and SO_2, and every 0.1 mg/m^3 increase in CO in working-age subgroup.

Table S6. Changes in the excess risk of pulmonary tuberculosis incidence and their 95% CIs for every 10 μg/m^3 increase in O_X, $PM_{2.5}$ and SO_2, and every 0.1 mg/m^3 increase in CO in elderly subgroup.

Table S7. Changes in the excess risk of pulmonary tuberculosis incidence and their 95% CIs for every 10 μg/m^3 increase in O_X, $PM_{2.5}$ and SO_2, and every 0.1 mg/m^3 increase in CO in lower NDVI areas.

Table S8. Changes in the excess risk of pulmonary tuberculosis incidence and their 95% CIs for every 10 μg/m^3 increase in O_X, $PM_{2.5}$ and SO_2, and every 0.1 mg/m^3 increase in CO in higher NDVI areas.

Figure S1. Time series of daily pulmonary tuberculosis incidence and air pollutant level averaged from 2013 to 2019. The red lines represented the median of daily PTB incidence and concentrations of air pollutants and the black lines (top and bottom) represented the IQR (25%, 75%).

Figure S2. Spearman rank correlation coefficients among air pollutant concentrations and meteorological data.

Figure S3. Effects of air pollutants on the risk of pulmonary tuberculosis incidence in specific and cumulative lag times when changing the maximum lag period (20 or 30 weeks).

Figure S4. Effects of air pollutants on the risk of pulmonary tuberculosis incidence in specific and cumulative lag times when changing the parameter settings of the degree of freedom (2 or 4).

Figure S5. Effects of air pollutants on the risk of pulmonary tuberculosis incidence in specific and cumulative lag times after adjusting for seasonal factors as covariates in the model.

Table S1. The average scores of Quasi-Akaike Information Criterion for the maximum lag period in 90 counties of Zhejiang Province, China, from 2013 to 2019.

The longest lag time	$PM_{2.5}$	CO	O_X	SO_2
16	1454.04	1451.81	1454.48	1454.62
17	1448.94	1447.32	1449.27	1449.30
18	1444.22	1442.51	1443.91	1444.38
19	1439.44	1437.79	1439.70	1439.99
20	1435.10	1434.05	1435.05	1435.67
21	1431.26	1430.07	1430.45	1431.41
22	1427.12	1425.99	1426.70	1427.41
23	1423.35	1422.32	1422.41	1422.80
24	1418.10	1417.22	1417.53	1418.19
25	1413.89	1413.10	1413.60	1414.41
**26**	**1409.50**	**1409.31**	**1409.38**	**1410.44**

Abbreviations: $PM_{2.5}$, particulate matter with an aerodynamic diameter of 2.5 µm or less; CO, carbon monoxide; O_X, the combined oxidant capacity; SO_2, Sulfur dioxide.

Table S2. Changes in the excess risk of pulmonary tuberculosis incidence and their 95% CIs for every 10 μg/m^3 increase in O_X, $PM_{2.5}$ and SO_2, and every 0.1 mg/m^3 increase in CO in Zhejiang Province, China, from 2013 to 2019.

lag time	$PM_{2.5}$		CO		O_X		SO_2	
	Specific lag time	Cumulative lag time	Specific lag time	Cumulative lag time	Specific lag time	Cumulative lag time	Specific lag time	Cumulative lag time
**Single-factor model [a]**								
0	-1.4(-3.2,0.4)	-1.4(-3.2,0.4)	-0.3(-2,1.3)	-0.3(-2,1.3)	0.8(-0.6,2.1)	0.8(-0.6,2.1)	-0.5(-1.9,1)	-0.5(-1.9,1)
1	-1.2(-2.7,0.2)	-2.6(-5.8,0.6)	-0.2(-1.6,1.1)	-0.6(-3.6,2.4)	0.6(-0.6,1.7)	1.3(-1.3,3.9)	-0.7(-1.8,0.5)	-1.1(-3.7,1.4)
2	-1(-2.2,0.1)	-3.7(-8,0.7)	-0.1(-1.2,0.9)	-0.7(-4.8,3.4)	0.4(-0.6,1.4)	1.7(-1.9,5.2)	-0.8(-1.8,0.1)	-2(-5.4,1.5)
3	-0.8(-1.7,0.1)	-4.6(-9.7,0.6)	0(-0.9,0.8)	-0.8(-5.7,4.1)	0.2(-0.7,1)	1.8(-2.6,6.2)	-1(-1.7,-0.3)	-3(-7.1,1.2)
4	-0.6(-1.3,0.1)	-5.2(-11,0.6)	0.1(-0.6,0.8)	-0.7(-6.2,4.8)	0(-0.7,0.7)	1.8(-3.2,6.9)	-1.2(-1.8,-0.5)	-4.1(-8.7,0.5)
5	-0.4(-1,0.2)	-5.7(-11.9,0.5)	0.1(-0.5,0.8)	-0.6(-6.5,5.3)	-0.1(-0.8,0.5)	1.7(-3.9,7.3)	-1.2(-1.8,-0.7)	-5.4(-10.3,-0.4)
6	-0.3(-1,0.4)	-5.9(-12.4,0.5)	0.2(-0.5,0.9)	-0.3(-6.5,5.9)	-0.3(-1,0.4)	1.4(-4.7,7.4)	-1.3(-1.9,-0.6)	-6.6(-11.9,-1.4)
7	-0.2(-1,0.6)	-5.9(-12.5,0.7)	0.3(-0.4,1)	0(-6.4,6.4)	-0.4(-1.1,0.2)	0.9(-5.5,7.3)	-1.2(-1.9,-0.5)	-7.9(-13.3,-2.4)
8	0(-0.9,0.8)	-5.7(-12.4,1.1)	0.3(-0.4,1.1)	0.5(-6.1,7.1)	-0.5(-1.2,0.1)	0.4(-6.4,7.2)	-1.1(-1.8,-0.4)	-9(-14.6,-3.4)
9	0.2(-0.7,1)	-5.2(-12.1,1.6)	0.4(-0.3,1.2)	1(-5.8,7.8)	-0.6(-1.3,0)	-0.2(-7.4,6.9)	-0.8(-1.6,-0.1)	-9.9(-15.7,-4.1)
10	0.4(-0.4,1.2)	-4.6(-11.6,2.4)	0.5(-0.2,1.3)	1.7(-5.3,8.7)	-0.7(-1.3,-0.01)	-0.9(-8.4,6.5)	-0.5(-1.2,0.1)	-10.5(-16.5,-4.5)
11	0.6(-0.1,1.3)	-3.9(-11,3.3)	0.6(-0.1,1.4)	2.5(-4.8,9.7)	-0.7(-1.4,-0.1)	-1.7(-9.5,6.1)	-0.1(-0.7,0.4)	-10.7(-17,-4.5)
12	0.8(0.2,1.4)	-3(-10.3,4.2)	0.7(-0.05,1.4)	3.3(-4.2,10.8)	-0.7(-1.4,-0.1)	-2.5(-10.7,5.7)	0.3(-0.2,0.8)	-10.5(-16.9,-4.1)
13	0.9(0.4,1.5)	-2.1(-9.5,5.3)	0.7(0.05,1.4)	4.4(-3.4,12.1)	-0.8(-1.4,-0.2)	-3.3(-11.9,5.3)	0.7(0.3,1.2)	-9.9(-16.5,-3.2)
14	1.1(0.5,1.7)	-1(-8.6,6.5)	0.7(-0.1,1.3)	5.5(-2.5,13.5)	-0.8(-1.4,-0.2)	-4.1(-13,4.9)	1.1(0.6,1.6)	-8.8(-15.6,-2)
15	1.3(0.6,2)	0(-7.7,7.8)	0.7(-0,1.4)	6.6(-1.6,14.9)	-0.9(-1.5,-0.3)	-4.9(-14.2,4.4)	1.4(0.9,1.9)	-7.3(-14.3,-0.4)
16	1.5(0.6,2.3)	1.1(-6.8,9.1)	0.7(-0,1.4)	7.8(-0.8,16.4)	-0.9(-1.5,-0.3)	-5.7(-15.4,4)	1.7(1.1,2.2)	-5.6(-12.7,1.5)
17	1.6(0.7,2.5)	2.3(-6,10.6)	0.7(-0.1,1.5)	8.9(-0.2,17.9)	-0.9(-1.5,-0.2)	-6.5(-16.6,3.6)	1.8(1.2,2.5)	-3.7(-10.9,3.6)
18	1.7(0.7,2.6)	3.6(-5.2,12.3)	0.7(-0.1,1.5)	9.8(0.3,19.3)	-0.9(-1.5,-0.2)	-7.3(-17.8,3.3)	1.9(1.3,2.5)	-1.7(-9.1,5.8)
19	1.7(0.8,2.6)	5(-4.3,14.2)	0.7(0,1.5)	10.6(0.6,20.6)	-0.8(-1.5,-0.2)	-8(-19,3)	1.8(1.2,2.4)	0.3(-7.4,7.9)
20	1.6(0.8,2.5)	6.5(-3.3,16.3)	0.7(0,1.5)	11.4(0.8,21.9)	-0.8(-1.4,-0.1)	-8.7(-20.1,2.7)	1.7(1.1,2.2)	2(-5.9,9.9)
21	1.6(0.8,2.4)	8(-2.3,18.4)	0.7(-0.1,1.4)	12(1,23)	-0.7(-1.4,0)	-9.4(-21.2,2.5)	1.4(0.8,1.9)	3.4(-4.6,11.5)
22	1.6(0.7,2.4)	9.7(-1.3,20.6)	0.6(-0.2,1.3)	12.6(1.1,24)	-0.7(-1.5,0)	-10(-22.3,2.4)	1(0.4,1.6)	4.5(-3.8,12.7)
23	1.6(0.6,2.6)	11.3(-0.1,22.8)	0.5(-0.3,1.3)	13(1.2,24.9)	-0.7(-1.5,0.1)	-10.5(-23.3,2.3)	0.5(-0.2,1.2)	5.1(-3.3,13.4)
24	1.6(0.4,2.8)	13.1(1,25.2)	0.5(-0.4,1.5)	13.4(1.1,25.7)	-0.7(-1.6,0.3)	-10.9(-24.2,2.4)	0.1(-0.8,1)	5.1(-3.4,13.6)
25	1.5(0,3.1)	14.9(2.2,27.7)	0.6(-0.6,1.7)	13.5(0.7,26.3)	-0.6(-1.8,0.5)	-11.3(-25.2,2.6)	-0.4(-1.6,0.7)	4.6(-4.2,13.4)
26	1.4(-0.5,3.3)	16.9(3.3,30.5)	0.6(-0.9,2)	13.6(0.1,27)	-0.6(-1.9,0.7)	-11.6(-26.2,3)	-1(-2.3,0.4)	3.5(-5.7,12.6)
**Multi-factor model [b]**								
0	-1.4(-3.4,0.6)	-1.4(-3.4,0.6)	-0.4(-2.1,1.3)	-0.4(-2.1,1.3)	0.1(-1.4,1.6)	0.1(-1.4,1.6)	0.5(-1,2.1)	0.5(-1,2.1)
1	-1.3(-2.9,0.3)	-2.7(-6.3,0.9)	-0.3(-1.7,1.1)	-0.7(-3.8,2.4)	0(-1.4,1.3)	0.1(-2.8,2.9)	0.2(-1.1,1.5)	0.7(-2.2,3.6)
2	-1.1(-2.4,0.2)	-3.9(-8.8,1.1)	-0.2(-1.3,0.9)	-0.9(-5.1,3.3)	-0.2(-1.3,0.9)	-0.1(-4.1,3.9)	-0.2(-1.2,0.8)	0.5(-3.4,4.4)
3	-1(-1.9,0)	-4.9(-10.7,1)	-0.1(-1,0.8)	-1(-6,4.1)	-0.3(-1.3,0.6)	-0.4(-5.3,4.5)	-0.5(-1.4,0.3)	0(-4.7,4.6)
4	-0.7(-1.5,0)	-5.7(-12.2,0.8)	0(-0.8,0.7)	-1(-6.7,4.7)	-0.4(-1.3,0.4)	-0.9(-6.5,4.8)	-0.9(-1.5,-0.2)	-0.9(-6.1,4.4)
5	-0.5(-1.2,0.1)	-6.3(-13.2,0.6)	0.1(-0.6,0.8)	-0.9(-7.1,5.2)	-0.5(-1.3,0.2)	-1.4(-7.7,4.8)	-1.2(-1.8,-0.5)	-1.9(-7.6,3.7)
6	-0.4(-1.2,0.3)	-6.8(-13.9,0.4)	0.2(-0.6,0.9)	-0.8(-7.2,5.7)	-0.6(-1.3,0.1)	-2.1(-8.8,4.7)	-1.4(-2,-0.7)	-3.2(-9.1,2.7)
7	-0.4(-1.2,0.5)	-7(-14.3,0.3)	0.2(-0.6,1)	-0.5(-7.3,6.3)	-0.7(-1.4,0.1)	-2.8(-10,4.4)	-1.5(-2.2,-0.8)	-4.5(-10.6,1.6)
8	-0.3(-1.2,0.6)	-7(-14.4,0.4)	0.3(-0.5,1.1)	-0.2(-7.2,6.8)	-0.7(-1.4,0)	-3.6(-11.1,4)	-1.4(-2.2,-0.7)	-5.9(-12.2,0.4)
9	-0.1(-1,0.8)	-6.8(-14.3,0.6)	0.4(-0.5,1.2)	0.2(-7.1,7.5)	-0.7(-1.4,0)	-4.4(-12.3,3.5)	-1.3(-2,-0.5)	-7.2(-13.6,-0.7)
10	0(-0.8,0.9)	-6.5(-14,1)	0.5(-0.4,1.3)	0.7(-6.9,8.3)	-0.7(-1.4,0)	-5.2(-13.5,3)	-1(-1.6,-0.3)	-8.2(-14.8,-1.5)
11	0.2(-0.6,1)	-6.1(-13.7,1.5)	0.5(-0.2,1.3)	1.3(-6.6,9.3)	-0.7(-1.4,0)	-6(-14.6,2.6)	-0.6(-1.2,0.1)	-8.8(-15.7,-2)
12	0.3(-0.3,1)	-5.6(-13.4,2.1)	0.5(-0.2,1.3)	2.1(-6.2,10.3)	-0.7(-1.3,0)	-6.8(-15.8,2.1)	-0.1(-0.6,0.5)	-9(-16,-2)

13	0.4(-0.2,1.1)	-5.1(-12.9,2.8)	0.5(-0.2,1.2)	2.9(-5.7,11.5)	-0.7(-1.3,0)	-7.6(-16.9,1.7)	0.4(-0.1,0.9)	-8.8(-15.9,-1.6)
14	0.5(-0.2,1.3)	-4.5(-12.5,3.5)	0.4(-0.3,1.1)	3.8(-5.1,12.7)	-0.7(-1.3,0)	-8.3(-18,1.4)	0.9(0.3,1.4)	-8(-15.3,-0.7)
15	0.7(-0.1,1.5)	-3.9(-12.1,4.3)	0.3(-0.4,1.1)	4.7(-4.4,13.9)	-0.7(-1.4,0)	-9(-19,1.1)	1.2(0.7,1.8)	-6.8(-14.2,0.7)
16	0.8(-0.1,1.7)	-3.3(-11.8,5.2)	0.3(-0.5,1.1)	5.7(-3.9,15.2)	-0.7(-1.4,0)	-9.6(-20.1,0.8)	1.5(0.9,2.2)	-5.2(-12.8,2.5)
17	0.9(-0.1,1.9)	-2.7(-11.6,6.2)	0.3(-0.5,1.2)	6.5(-3.4,16.4)	-0.7(-1.5,0)	-10.2(-21.1,0.6)	1.7(1.1,2.4)	-3.3(-11.1,4.5)
18	1(0,2)	-1.9(-11.4,7.5)	0.4(-0.5,1.2)	7.2(-3.1,17.5)	-0.7(-1.5,0)	-10.8(-22.1,0.5)	1.8(1.1,2.5)	-1.3(-9.4,6.7)
19	1(0.1,2)	-1.1(-11.1,9)	0.4(-0.4,1.3)	7.8(-2.9,18.5)	-0.7(-1.4,0)	-11.4(-23.2,0.4)	1.8(1.1,2.4)	0.6(-7.7,8.9)
20	1.1(0.2,2)	-0.1(-10.8,10.6)	0.5(-0.3,1.3)	8.3(-2.9,19.5)	-0.7(-1.4,0)	-12(-24.2,0.3)	1.6(1,2.2)	2.3(-6.2,10.8)
21	1.1(0.3,2)	1.1(-10.3,12.4)	0.5(-0.4,1.3)	8.7(-2.9,20.4)	-0.7(-1.5,0)	-12.6(-25.3,0.2)	1.3(0.7,1.9)	3.7(-5.1,12.4)
22	1.3(0.3,2.2)	2.4(-9.6,14.3)	0.4(-0.5,1.2)	9.1(-3,21.3)	-0.8(-1.5,0)	-13.2(-26.4,0.1)	0.9(0.3,1.5)	4.6(-4.3,13.5)
23	1.4(0.4,2.4)	3.8(-8.8,16.4)	0.4(-0.6,1.3)	9.5(-3.1,22.1)	-0.8(-1.7,0)	-13.8(-27.5,0)	0.4(-0.3,1.2)	5.1(-4,14.1)
24	1.4(0.2,2.7)	5.4(-7.9,18.6)	0.5(-0.6,1.5)	9.7(-3.4,22.8)	-0.9(-1.8,0)	-14.4(-28.7,-0.1)	-0.1(-1,0.8)	4.9(-4.3,14.2)
25	1.4(-0.2,3)	7.1(-6.8,21.1)	0.6(-0.6,1.9)	9.8(-3.9,23.5)	-0.9(-2,0.1)	-15(-29.9,-0.2)	-0.6(-1.8,0.5)	4.1(-5.3,13.6)
26	1.4(-0.6,3.3)	9.1(-5.8,24)	0.7(-0.8,2.2)	9.9(-4.5,24.3)	-1(-2.2,0.3)	-15.7(-31.2,-0.2)	-1.2(-2.6,0.2)	2.7(-7.2,12.5)

[a]: Adjusted for WS, AT, RH, SSD and TP;

[b]: Adjusted for WS, AT, RH, SSD, TP, and other air pollutants which not in cross-basis;

Abbreviations: $PM_{2.5}$, particulate matter with an aerodynamic diameter of 2.5 μm or less; CO, carbon monoxide; O_X, the combined oxidant capacity; SO_2, Sulfur dioxide; AT, average temperature; WS, wind speed; RH, relative humidity; SSD, sunshine duration; TP, total precipitation.

Table S3. Changes in the excess risk of pulmonary tuberculosis incidence and their 95% CIs for every 10 μg/m^3 increase in O_X, $PM_{2.5}$ and SO_2, and every 0.1 mg/m^3 increase in CO in male subgroup.

lag time	$PM_{2.5}$		CO		O_X		SO_2	
	Specific lag time	Cumulative lag time	Specific lag time	Cumulative lag time	Specific lag time	Cumulative lag time	Specific lag time	Cumulative lag time
**Single-factor model [a]**								
0	-0.4(-2.5,1.7)	-0.4(-2.5,1.7)	-0.3(-2.2,1.5)	-0.3(-2.2,1.5)	1.6(-0.03,3.2)	1.6(-0.03,3.2)	-1.7(-3.3,-0.01)	-1.7(-3.3,-0.01)
1	-0.3(-2,1.4)	-0.7(-4.5,3.1)	-0.2(-1.7,1.4)	-0.5(-4,2.9)	1.2(-0.2,2.6)	2.8(-0.2,5.7)	-1.5(-2.9,-0.2)	-3.2(-6.2,-0.2)
2	-0.3(-1.6,1.1)	-1(-6.1,4.1)	0(-1.3,1.2)	-0.6(-5.2,4.1)	0.9(-0.3,2)	3.6(-0.5,7.7)	-1.4(-2.5,-0.4)	-4.6(-8.7,-0.6)
3	-0.2(-1.2,0.9)	-1.3(-7.4,4.8)	0.1(-0.9,1.1)	-0.4(-6.1,5.2)	0.5(-0.4,1.5)	4.2(-0.9,9.2)	-1.3(-2.2,-0.5)	-5.9(-10.8,-1.1)
4	-0.1(-0.9,0.8)	-1.4(-8.2,5.4)	0.3(-0.6,1.1)	-0.2(-6.5,6.2)	0.2(-0.6,1.1)	4.4(-1.4,10.2)	-1.2(-1.9,-0.5)	-7.1(-12.5,-1.7)
5	0(-0.7,0.8)	-1.4(-8.7,5.8)	0.4(-0.4,1.2)	0.2(-6.7,7.1)	0(-0.8,0.7)	4.3(-2.1,10.7)	-1.1(-1.8,-0.4)	-8.2(-14,-2.3)
6	0.1(-0.7,0.9)	-1.3(-8.9,6.2)	0.5(-0.4,1.3)	0.8(-6.5,8.1)	-0.3(-1,0.5)	4(-3,10.9)	-0.9(-1.7,-0.1)	-9.1(-15.2,-2.9)
7	0.1(-0.8,1)	-1(-8.8,6.7)	0.5(-0.3,1.4)	1.4(-6.3,9)	-0.5(-1.2,0.3)	3.5(-3.9,10.8)	-0.7(-1.5,0.1)	-9.8(-16.1,-3.4)
8	0.2(-0.7,1.2)	-0.5(-8.4,7.4)	0.6(-0.3,1.5)	2.1(-5.9,10)	-0.6(-1.4,0.1)	2.8(-5,10.6)	-0.4(-1.3,0.4)	-10.3(-16.9,-3.7)
9	0.4(-0.6,1.3)	0.1(-7.9,8.2)	0.7(-0.2,1.5)	2.9(-5.4,11.2)	-0.8(-1.5,0.03)	2(-6.2,10.2)	-0.2(-1,0.6)	-10.5(-17.4,-3.7)
10	0.6(-0.3,1.5)	0.9(-7.3,9.2)	0.7(-0.1,1.6)	3.7(-5,12.3)	-0.8(-1.6,-0.08)	1.1(-7.5,9.8)	0.1(-0.6,0.8)	-10.5(-17.6,-3.4)
11	0.8(0.07,1.6)	1.9(-6.5,10.3)	0.8(-0.01,1.6)	4.6(-4.4,13.6)	-0.8(-1.5,-0.1)	0.3(-8.8,9.3)	0.4(-0.2,1.1)	-10.2(-17.6,-2.8)
12	1(0.4,1.6)	3(-5.6,11.5)	0.8(0.1,1.5)	5.5(-3.8,14.9)	-0.8(-1.5,-0.1)	-0.6(-10.1,8.9)	0.8(0.2,1.3)	-9.6(-17.3,-1.9)
13	1.2(0.5,1.8)	4.2(-4.5,12.9)	0.7(-0.1,1.5)	6.6(-3.1,16.2)	-0.8(-1.5,-0.1)	-1.4(-11.3,8.5)	1.1(0.6,1.6)	-8.6(-16.5,-0.7)
14	1.3(0.6,2.1)	5.5(-3.4,14.3)	0.7(-0.1,1.4)	7.6(-2.3,17.6)	-0.8(-1.6,-0.1)	-2.2(-12.5,8.1)	1.3(0.8,1.9)	-7.3(-15.4,0.8)
15	1.5(0.7,2.4)	6.8(-2.2,15.9)	0.6(-0.2,1.4)	8.7(-1.6,19)	-0.8(-1.6,-0.1)	-2.9(-13.7,7.8)	1.6(1,2.2)	-5.7(-13.9,2.4)
16	1.7(0.7,2.7)	8.2(-1.1,17.6)	0.6(-0.4,1.5)	9.7(-0.9,20.3)	-0.8(-1.6,-0.03)	-3.6(-14.9,7.6)	1.7(1.1,2.4)	-4(-12.2,4.3)
17	1.8(0.7,2.9)	9.7(-0.1,19.5)	0.5(-0.5,1.5)	10.6(-0.4,21.7)	-0.9(-1.7,-0.05)	-4.3(-16,7.5)	1.8(1.1,2.6)	-2.1(-10.6,6.4)
18	1.8(0.7,2.9)	11.2(0.9,21.5)	0.5(-0.5,1.5)	11.4(-0.1,23)	-0.8(-1.6,-0.01)	-5(-17.2,7.3)	1.8(1.1,2.6)	-0.1(-8.9,8.6)
19	1.8(0.7,2.8)	12.8(1.9,23.6)	0.5(-0.4,1.4)	12.1(0,24.1)	-0.8(-1.6,-0.03)	-5.6(-18.5,7.2)	1.8(1.1,2.5)	1.7(-7.3,10.7)
20	1.7(0.7,2.6)	14.3(2.8,25.8)	0.5(-0.3,1.4)	12.6(-0.1,25.2)	-0.8(-1.6,-0.08)	-6.3(-19.7,7)	1.6(1,2.3)	3.5(-5.8,12.7)
21	1.5(0.6,2.4)	15.9(3.7,28)	0.5(-0.4,1.3)	13(-0.2,26.2)	-0.8(-1.6,-0.09)	-7.1(-21,6.8)	1.4(0.8,2)	4.9(-4.5,14.4)
22	1.5(0.5,2.4)	17.3(4.5,30.1)	0.4(-0.5,1.3)	13.3(-0.5,27.1)	-1(-1.8,-0.2)	-7.9(-22.3,6.6)	1.1(0.4,1.8)	6.1(-3.5,15.8)
23	1.4(0.3,2.5)	18.7(5.3,32.2)	0.4(-0.7,1.4)	13.6(-0.8,27.9)	-1.1(-2,-0.2)	-8.7(-23.7,6.3)	0.8(-0.05,1.6)	7(-2.9,16.8)
24	1.3(-0.1,2.6)	20.1(6,34.2)	0.4(-0.8,1.6)	13.7(-1.3,28.7)	-1.1(-2.2,-0.1)	-9.6(-25.1,6)	0.4(-0.6,1.4)	7.5(-2.6,17.5)
25	1(-0.7,2.8)	21.4(6.5,36.3)	0.5(-0.9,1.9)	13.7(-2,29.4)	-1.2(-2.5,0.1)	-10.6(-26.8,5.7)	0(-1.3,1.3)	7.5(-2.8,17.8)
26	0.8(-1.4,3)	22.7(6.8,38.6)	0.6(-1.1,2.3)	13.6(-3,30.2)	-1.2(-2.8,0.3)	-11.6(-28.6,5.4)	-0.4(-2,1.3)	7.1(-3.6,17.9)
**Multi-factor model [b]**								
0	0.2(-2.2,2.5)	0.2(-2.2,2.5)	-0.4(-2.4,1.6)	-0.4(-2.4,1.6)	0.6(-1.2,2.4)	0.6(-1.2,2.4)	-0.3(-2.2,1.5)	-0.3(-2.2,1.5)
1	0.1(-1.8,2)	0.2(-4,4.5)	-0.2(-1.9,1.4)	-0.6(-4.3,3)	0.3(-1.2,1.9)	0.9(-2.5,4.4)	-0.5(-2,1.1)	-0.8(-4.1,2.6)
2	0(-1.5,1.5)	0.2(-5.5,6)	0(-1.4,1.3)	-0.7(-5.6,4.3)	0.1(-1.2,1.5)	1(-3.7,5.8)	-0.6(-1.8,0.6)	-1.3(-5.9,3.2)
3	0(-1.2,1.2)	0.2(-6.7,7)	0.1(-1,1.2)	-0.5(-6.5,5.4)	-0.1(-1.2,1)	0.9(-4.9,6.8)	-0.7(-1.7,0.2)	-2(-7.5,3.4)
4	0(-0.9,1)	0.1(-7.5,7.7)	0.3(-0.6,1.2)	-0.3(-7,6.5)	-0.3(-1.3,0.6)	0.6(-6.1,7.4)	-0.9(-1.7,-0.1)	-2.8(-9,3.3)
5	0.1(-0.7,0.9)	0.1(-8.1,8.2)	0.5(-0.4,1.3)	0.2(-7.1,7.5)	-0.5(-1.4,0.4)	0.1(-7.4,7.6)	-1(-1.7,-0.3)	-3.7(-10.3,2.9)
6	0.1(-0.8,0.9)	0.1(-8.3,8.5)	0.6(-0.3,1.5)	0.7(-7.1,8.5)	-0.6(-1.5,0.2)	-0.6(-8.6,7.5)	-1.1(-1.8,-0.4)	-4.7(-11.6,2.3)
7	0(-1,1)	0.2(-8.4,8.7)	0.7(-0.3,1.6)	1.3(-6.8,9.5)	-0.7(-1.6,0.1)	-1.4(-9.9,7.2)	-1.1(-1.8,-0.3)	-5.6(-12.8,1.5)
8	0(-1,1.1)	0.4(-8.3,9.1)	0.7(-0.3,1.7)	2(-6.5,10.6)	-0.8(-1.6,0)	-2.3(-11.3,6.7)	-1(-1.8,-0.1)	-6.6(-13.9,0.8)
9	0.1(-1,1.1)	0.7(-8,9.5)	0.7(-0.2,1.7)	2.8(-6.1,11.7)	-0.8(-1.6,0)	-3.2(-12.6,6.2)	-0.8(-1.6,0)	-7.4(-15,0.2)
10	0.1(-0.8,1.1)	1.1(-7.7,10)	0.7(-0.2,1.6)	3.7(-5.6,12.9)	-0.8(-1.6,0)	-4.1(-14,5.7)	-0.5(-1.2,0.3)	-8(-15.8,-0.2)
11	0.2(-0.6,1.1)	1.6(-7.4,10.5)	0.7(-0.2,1.5)	4.5(-5.2,14.2)	-0.7(-1.5,0)	-5(-15.2,5.2)	-0.1(-0.8,0.5)	-8.2(-16.3,-0.2)
12	0.3(-0.5,1.1)	2.1(-7,11.2)	0.6(-0.2,1.4)	5.3(-4.7,15.3)	-0.7(-1.4,0.1)	-5.8(-16.5,4.8)	0.2(-0.3,0.8)	-8.2(-16.4,0.1)
13	0.4(-0.4,1.2)	2.6(-6.6,11.8)	0.4(-0.3,1.2)	6.1(-4.2,16.5)	-0.6(-1.3,0.1)	-6.5(-17.6,4.6)	0.6(0.1,1.2)	-7.7(-16.1,0.7)

14	0.5(-0.4,1.4)	3.1(-6.3,12.5)	0.2(-0.6,1.1)	6.9(-3.8,17.6)	-0.6(-1.3,0.2)	-7.1(-18.6,4.4)	1(0.4,1.6)	-6.8(-15.4,1.8)
15	0.6(-0.4,1.6)	3.6(-6,13.3)	0.1(-0.8,1)	7.5(-3.5,18.6)	-0.5(-1.4,0.3)	-7.6(-19.6,4.3)	1.3(0.6,2)	-5.6(-14.3,3.2)
16	0.7(-0.5,1.9)	4.2(-5.8,14.1)	0(-1,1)	8.1(-3.3,19.5)	-0.5(-1.4,0.3)	-8.1(-20.5,4.4)	1.5(0.8,2.3)	-4(-12.9,5)
17	0.8(-0.5,2)	4.7(-5.7,15.2)	-0.1(-1.1,1)	8.5(-3.3,20.3)	-0.5(-1.4,0.4)	-8.5(-21.5,4.5)	1.7(0.9,2.5)	-2.2(-11.3,7)
18	0.8(-0.5,2.1)	5.3(-5.8,16.4)	-0.1(-1.1,1)	8.7(-3.6,21)	-0.5(-1.4,0.3)	-8.9(-22.4,4.7)	1.7(0.9,2.5)	-0.2(-9.7,9.2)
19	0.8(-0.4,2)	6(-5.8,17.8)	0(-1,1)	8.7(-4.1,21.6)	-0.6(-1.4,0.3)	-9.3(-23.5,4.9)	1.7(0.9,2.5)	1.6(-8.1,11.3)
20	0.7(-0.4,1.8)	6.7(-5.9,19.2)	0.1(-0.9,1)	8.7(-4.7,22.1)	-0.6(-1.5,0.2)	-9.8(-24.6,5)	1.5(0.8,2.3)	3.3(-6.7,13.3)
21	0.7(-0.4,1.7)	7.4(-5.9,20.8)	0.1(-0.9,1)	8.7(-5.3,22.7)	-0.8(-1.6,0.1)	-10.3(-25.7,5.1)	1.3(0.6,2)	4.7(-5.6,14.9)
22	0.7(-0.3,1.8)	8.2(-5.9,22.3)	0.1(-0.9,1.1)	8.7(-5.9,23.3)	-0.9(-1.8,-0.1)	-11(-27,5)	1(0.2,1.7)	5.7(-4.8,16.2)
23	0.8(-0.4,2)	9(-5.8,23.8)	0.2(-0.9,1.3)	8.7(-6.5,23.9)	-1.1(-2,-0.2)	-11.8(-28.4,4.9)	0.6(-0.3,1.4)	6.3(-4.3,17)
24	0.8(-0.7,2.2)	9.8(-5.7,25.4)	0.4(-0.9,1.6)	8.8(-7.1,24.6)	-1.3(-2.3,-0.2)	-12.7(-30,4.6)	0.2(-0.9,1.2)	6.5(-4.3,17.3)
25	0.6(-1.2,2.4)	10.7(-5.7,27.1)	0.7(-0.8,2.1)	8.8(-7.8,25.5)	-1.4(-2.6,-0.1)	-13.7(-31.7,4.3)	-0.3(-1.6,1.1)	6.1(-5,17.2)
26	0.4(-1.8,2.7)	11.7(-5.7,29.1)	0.9(-0.9,2.7)	9(-8.5,26.5)	-1.5(-3,0)	-14.9(-33.7,3.9)	-0.7(-2.4,1)	5.1(-6.4,16.7)

[a]*:* Adjusted for WS, AT, RH, SSD and TP;

[b]*:* Adjusted for WS, AT, RH, SSD, TP, and other air pollutants which not in cross-basis;

Abbreviations: $PM_{2.5}$, particulate matter with an aerodynamic diameter of 2.5 μm or less; CO, carbon monoxide; O_X, the combined oxidant capacity; SO_2, Sulfur dioxide; AT, average temperature; WS, wind speed; RH, relative humidity; SSD, sunshine duration; TP, total precipitation.

Table S4. Changes in the excess risk of pulmonary tuberculosis incidence and their 95% CIs for every 10 μg/m^3 increase in O_X, $PM_{2.5}$ and SO_2, and every 0.1 mg/m^3 increase in CO in female subgroup.

lag time	$PM_{2.5}$		CO		O_X		SO_2	
	Specific lag time	Cumulative lag time	Specific lag time	Cumulative lag time	Specific lag time	Cumulative lag time	Specific lag time	Cumulative lag time
**Single-factor model [a]**								
0	-3.4(-6.7,-0.1)	-3.4(-6.7,-0.1)	-0.7(-3.3,2)	-0.7(-3.3,2)	-1.3(-3.8,1.1)	-1.3(-3.8,1.1)	2.6(-0.1,5.2)	2.6(-0.1,5.2)
1	-3(-5.7,-0.3)	-6.4(-12.4,-0.4)	-0.7(-2.9,1.5)	-1.4(-6.2,3.5)	-1.1(-3.2,0.9)	-2.5(-7,2)	1.6(-0.5,3.8)	4.2(-0.7,9)
2	-2.6(-4.8,-0.5)	-9.1(-17.2,-1)	-0.7(-2.4,1)	-2.1(-8.6,4.4)	-0.9(-2.7,0.8)	-3.4(-9.7,2.8)	0.7(-1,2.4)	4.9(-1.6,11.4)
3	-2.2(-3.9,-0.6)	-11.3(-21,-1.7)	-0.7(-2,0.7)	-2.8(-10.6,5)	-0.7(-2.2,0.8)	-4.1(-11.8,3.5)	-0.2(-1.4,1.1)	4.7(-2.9,12.4)
4	-1.8(-3.1,-0.5)	-13.2(-24,-2.5)	-0.6(-1.7,0.5)	-3.5(-12.1,5.1)	-0.5(-1.7,0.8)	-4.7(-13.5,4.2)	-0.9(-2,0.1)	3.8(-4.6,12.2)
5	-1.5(-2.7,-0.3)	-14.8(-26.2,-3.3)	-0.5(-1.6,0.5)	-4.1(-13.2,5.1)	-0.3(-1.5,0.8)	-4.9(-14.7,4.8)	-1.6(-2.6,-0.6)	2.2(-6.6,11)
6	-1.2(-2.5,0)	-15.9(-27.8,-4.1)	-0.5(-1.6,0.6)	-4.5(-13.9,4.9)	-0.3(-1.4,0.8)	-5(-15.6,5.5)	-2.1(-3.1,-1.1)	0.1(-9,9.1)
7	-1(-2.4,0.3)	-16.7(-28.9,-4.6)	-0.4(-1.6,0.8)	-4.8(-14.7,5.1)	-0.3(-1.4,0.9)	-5(-16.2,6.2)	-2.4(-3.5,-1.3)	-2.4(-11.8,7)
8	-0.8(-2.2,0.7)	-17.2(-29.6,-4.9)	-0.3(-1.6,0.9)	-5(-15.3,5.3)	-0.3(-1.5,0.9)	-4.9(-16.6,6.8)	-2.5(-3.7,-1.4)	-5(-14.7,4.7)
9	-0.5(-1.9,0.9)	-17.5(-30.1,-4.9)	-0.2(-1.4,1.1)	-5(-15.7,5.8)	-0.3(-1.5,0.9)	-4.8(-17.1,7.4)	-2.5(-3.7,-1.2)	-7.5(-17.6,2.5)
10	-0.2(-1.5,1)	-17.6(-30.5,-4.6)	0(-1.1,1.2)	-4.8(-16,6.4)	-0.3(-1.4,0.8)	-4.9(-17.7,7.9)	-2.1(-3.3,-1)	-9.7(-20.1,0.7)
11	0(-1.1,1.1)	-17.5(-30.7,-4.2)	0.3(-0.9,1.4)	-4.4(-16.1,7.3)	-0.4(-1.4,0.7)	-5.1(-18.3,8.2)	-1.6(-2.6,-0.6)	-11.3(-22.1,-0.6)
12	0.2(-0.7,1.2)	-17.3(-30.8,-3.7)	0.5(-0.6,1.6)	-3.7(-15.9,8.5)	-0.5(-1.4,0.5)	-5.4(-19.2,8.4)	-0.9(-1.8,-0.1)	-12.3(-23.5,-1.2)
13	0.3(-0.6,1.2)	-16.9(-30.8,-3.1)	0.6(-0.5,1.7)	-2.8(-15.4,9.9)	-0.6(-1.6,0.3)	-5.8(-20.1,8.5)	-0.2(-1,0.6)	-12.6(-24.1,-1.2)
14	0.4(-0.5,1.4)	-16.5(-30.7,-2.3)	0.7(-0.5,1.9)	-1.5(-14.5,11.5)	-0.8(-1.7,0.2)	-6.4(-21.3,8.5)	0.5(-0.4,1.3)	-12.2(-23.9,-0.6)
15	0.6(-0.5,1.8)	-16(-30.5,-1.4)	0.9(-0.4,2.2)	0(-13.5,13.5)	-0.9(-1.9,0.1)	-7.1(-22.5,8.4)	1.1(0.2,2.1)	-11.1(-23,0.8)
16	0.8(-0.5,2.2)	-15.3(-30.3,-0.3)	1(-0.4,2.4)	1.7(-12.3,15.6)	-1(-2.1,0.1)	-7.8(-23.9,8.2)	1.7(0.6,2.7)	-9.5(-21.6,2.7)
17	1.1(-0.5,2.6)	-14.4(-30,1.1)	1.1(-0.3,2.6)	3.4(-11.1,18)	-1(-2.1,0.2)	-8.6(-25.4,8.1)	2(0.9,3.2)	-7.4(-19.9,5.1)
18	1.2(-0.3,2.8)	-13.4(-29.7,3)	1.2(-0.2,2.7)	5.2(-10,20.4)	-0.9(-2,0.3)	-9.4(-26.9,8.1)	2.2(1,3.4)	-5.2(-18,7.7)
19	1.4(-0.2,2.9)	-12.1(-29.3,5.2)	1.3(-0.1,2.7)	6.9(-9.1,22.9)	-0.7(-1.9,0.4)	-10.1(-28.3,8.2)	2.2(1,3.3)	-3(-16.2,10.2)
20	1.5(0.06,3)	-10.5(-28.7,7.7)	1.3(-0.01,2.6)	8.4(-8.3,25.2)	-0.5(-1.7,0.6)	-10.5(-29.6,8.5)	1.9(0.9,2.9)	-1(-14.8,12.8)
21	1.6(0.2,3.1)	-8.7(-27.9,10.5)	1.2(0.01,2.4)	9.8(-7.9,27.5)	-0.3(-1.4,0.8)	-10.7(-30.5,9.1)	1.5(0.6,2.5)	0.5(-13.8,14.9)
22	1.8(0.3,3.3)	-6.7(-27,13.5)	1.1(-0.1,2.3)	11.1(-7.3,29.5)	-0.1(-1.2,1.1)	-10.6(-31.1,9.9)	1(-0.1,2)	1.5(-13.2,16.3)
23	2(0.3,3.7)	-4.5(-25.8,16.8)	0.9(-0.4,2.3)	12.3(-6.7,31.3)	0.1(-1.2,1.5)	-10.1(-31.3,11.1)	0.3(-0.9,1.5)	1.8(-13.2,16.8)
24	2.3(0.2,4.4)	-2(-24.4,20.4)	0.7(-1,2.3)	13.4(-6,32.9)	0.3(-1.3,1.9)	-9.3(-31.2,12.6)	-0.5(-2,1)	1.2(-14,16.4)
25	2.5(0.01,5.1)	0.8(-22.9,24.4)	0.5(-1.6,2.5)	14.2(-5.9,34.3)	0.5(-1.4,2.3)	-8.1(-30.8,14.5)	-1.3(-3.2,0.6)	-0.3(-15.7,15.1)
26	2.7(-0.3,5.8)	3.8(-21.5,29.1)	0.3(-2.2,2.8)	14.6(-6.4,35.7)	0.7(-1.5,2.8)	-6.7(-30.3,16.8)	-2.1(-4.5,0.2)	-2.8(-18.7,13.1)
**Multi-factor model [b]**								
0	-4.6(-8.2,-1)	-4.6(-8.2,-1)	-0.9(-3.6,1.7)	-0.9(-3.6,1.7)	-1.4(-4,1.3)	-1.4(-4,1.3)	3.2(0.2,6.2)	3.2(0.2,6.2)
1	-4(-7,-1.1)	-8.7(-15.1,-2.2)	-0.9(-3.1,1.2)	-1.9(-6.8,3)	-1.2(-3.5,1.1)	-2.6(-7.5,2.4)	2.1(-0.3,4.6)	5.3(-0.1,10.7)
2	-3.5(-5.8,-1.2)	-12.2(-20.9,-3.4)	-0.9(-2.7,0.9)	-2.8(-9.4,3.8)	-1(-2.9,0.9)	-3.6(-10.5,3.3)	1.1(-0.7,3)	6.4(-0.9,13.7)
3	-3(-4.8,-1.1)	-15.1(-25.6,-4.6)	-0.9(-2.3,0.6)	-3.7(-11.6,4.2)	-0.8(-2.4,0.8)	-4.4(-12.9,4)	0.2(-1.2,1.6)	6.6(-2,15.2)
4	-2.5(-4,-1)	-17.5(-29.3,-5.8)	-0.8(-2,0.3)	-4.5(-13.3,4.4)	-0.6(-2,0.7)	-5.1(-14.8,4.6)	-0.6(-1.8,0.5)	6(-3.5,15.5)
5	-2.1(-3.4,-0.7)	-19.5(-32.1,-6.9)	-0.9(-2,0.3)	-5.2(-14.8,4.4)	-0.5(-1.7,0.7)	-5.6(-16.4,5.1)	-1.4(-2.4,-0.3)	4.6(-5.4,14.6)
6	-1.7(-3,-0.3)	-21(-34.2,-7.8)	-0.8(-2,0.3)	-5.8(-16,4.3)	-0.4(-1.6,0.7)	-6(-17.6,5.6)	-2(-3.1,-0.9)	2.7(-7.6,12.9)
7	-1.2(-2.7,0.2)	-22.2(-35.8,-8.6)	-0.8(-2,0.5)	-6.4(-17,4.2)	-0.4(-1.6,0.8)	-6.2(-18.5,6)	-2.4(-3.5,-1.3)	0.3(-10.2,10.9)
8	-0.8(-2.3,0.7)	-23(-37,-9.1)	-0.6(-1.9,0.7)	-6.9(-17.9,4.1)	-0.4(-1.6,0.8)	-6.4(-19.3,6.4)	-2.6(-3.8,-1.4)	-2.2(-13,8.7)
9	-0.5(-1.9,1)	-23.6(-37.8,-9.4)	-0.4(-1.7,0.9)	-7.2(-18.7,4.2)	-0.4(-1.6,0.8)	-6.6(-20,6.7)	-2.5(-3.8,-1.3)	-4.6(-15.8,6.5)
10	-0.1(-1.5,1.2)	-23.9(-38.4,-9.3)	-0.1(-1.4,1.1)	-7.4(-19.3,4.6)	-0.5(-1.6,0.7)	-6.9(-20.8,7)	-2.2(-3.4,-1.1)	-6.8(-18.3,4.7)
11	0.2(-1,1.3)	-23.9(-38.7,-9)	0.2(-1,1.3)	-7.2(-19.6,5.3)	-0.5(-1.6,0.6)	-7.2(-21.6,7.2)	-1.7(-2.7,-0.7)	-8.5(-20.4,3.3)
12	0.3(-0.7,1.4)	-23.6(-38.7,-8.5)	0.4(-0.7,1.5)	-6.6(-19.5,6.3)	-0.6(-1.6,0.4)	-7.7(-22.6,7.2)	-1(-1.9,-0.1)	-9.6(-21.8,2.5)

13	0.4(-0.6,1.5)	-23.1(-38.4,-7.7)	0.6(-0.5,1.7)	-5.7(-19,7.7)	-0.7(-1.7,0.2)	-8.3(-23.7,7.1)	-0.3(-1.2,0.6)	-10.1(-22.5,2.3)
14	0.6(-0.6,1.7)	-22.3(-37.9,-6.6)	0.7(-0.5,1.9)	-4.3(-18,9.5)	-0.9(-1.9,0.1)	-8.9(-24.8,7)	0.4(-0.5,1.4)	-9.8(-22.4,2.9)
15	0.7(-0.6,2)	-21.3(-37.3,-5.4)	0.9(-0.4,2.2)	-2.5(-16.7,11.6)	-1(-2,0.1)	-9.7(-26.1,6.7)	1.1(0,2.1)	-8.8(-21.7,4.1)
16	0.9(-0.6,2.4)	-20.2(-36.5,-3.9)	1.1(-0.4,2.5)	-0.6(-15.3,14.1)	-1(-2.2,0.1)	-10.5(-27.4,6.5)	1.6(0.4,2.8)	-7.2(-20.4,6)
17	1.1(-0.5,2.8)	-18.9(-35.9,-2)	1.2(-0.3,2.7)	1.5(-13.8,16.8)	-1(-2.2,0.2)	-11.3(-28.9,6.3)	2(0.7,3.2)	-5.3(-18.8,8.2)
18	1.4(-0.3,3.1)	-17.5(-35.2,0.1)	1.3(-0.2,2.8)	3.5(-12.6,19.5)	-1(-2.2,0.2)	-12.1(-30.3,6.1)	2.1(0.9,3.4)	-3.1(-17.2,10.9)
19	1.6(0,3.3)	-15.9(-34.5,2.7)	1.4(-0.1,2.8)	5.3(-11.6,22.2)	-0.9(-2,0.3)	-12.8(-31.7,6)	2.1(0.9,3.3)	-0.9(-15.7,13.9)
20	1.9(0.3,3.5)	-14(-33.6,5.6)	1.4(0,2.8)	7(-10.7,24.7)	-0.7(-1.8,0.4)	-13.4(-33,6.1)	1.9(0.8,3)	1.1(-14.5,16.7)
21	2.2(0.6,3.7)	-11.8(-32.5,8.9)	1.4(0,2.7)	8.6(-9.9,27.1)	-0.5(-1.6,0.7)	-13.8(-34.1,6.4)	1.6(0.5,2.6)	2.7(-13.5,18.8)
22	2.4(0.8,4)	-9.3(-31.1,12.5)	1.2(-0.1,2.6)	10(-9.2,29.3)	-0.3(-1.5,0.9)	-14(-35,6.9)	1.1(0,2.1)	3.7(-12.9,20.3)
23	2.7(0.8,4.5)	-6.4(-29.3,16.5)	0.9(-0.6,2.5)	11.4(-8.5,31.3)	-0.1(-1.5,1.2)	-14(-35.6,7.7)	0.4(-0.9,1.7)	4(-12.9,21)
24	2.9(0.6,5.1)	-3.2(-27.3,20.9)	0.6(-1.2,2.5)	12.5(-8.1,33.1)	0(-1.6,1.6)	-13.6(-36.1,8.8)	-0.4(-1.9,1.2)	3.5(-13.6,20.7)
25	3(0.2,5.9)	0.4(-25.1,25.9)	0.3(-1.9,2.6)	13.3(-8.1,34.6)	0.1(-1.7,2)	-13(-36.3,10.3)	-1.2(-3.2,0.8)	2.1(-15.3,19.5)
26	3.2(-0.2,6.7)	4.2(-23,31.4)	0.1(-2.6,2.8)	13.6(-8.8,36)	0.3(-1.9,2.5)	-12.2(-36.5,12.1)	-2(-4.4,0.4)	-0.2(-18,17.5)

[a]: Adjusted for WS, AT, RH, SSD and TP;

[b]: Adjusted for WS, AT, RH, SSD, TP, and other air pollutants which not in cross-basis;

Abbreviations: $PM_{2.5}$, particulate matter with an aerodynamic diameter of 2.5 μm or less; CO, carbon monoxide; O_X, the combined oxidant capacity; SO_2, Sulfur dioxide; AT, average temperature; WS, wind speed; RH, relative humidity; SSD, sunshine duration; TP, total precipitation.

Table S5. Changes in the excess risk of pulmonary tuberculosis incidence and their 95% CIs for every 10 μg/m^3 increase in O_X, $PM_{2.5}$ and SO_2, and every 0.1 mg/m^3 increase in CO in working-age subgroup.

lag time	$PM_{2.5}$		CO		O_X		SO_2	
	Specific lag time	Cumulative lag time	Specific lag time	Cumulative lag time	Specific lag time	Cumulative lag time	Specific lag time	Cumulative lag time
**Single-factor model [a]**								
0	-1.2(-3.7,1.3)	-1.2(-3.7,1.3)	-0.2(-2.6,2.3)	-0.2(-2.6,2.3)	0.8(-0.8,2.5)	0.8(-0.8,2.5)	-0.4(-2.2,1.5)	-0.4(-2.2,1.5)
1	-1.1(-3.1,1)	-2.3(-6.8,2.3)	0(-2.1,2)	-0.2(-4.7,4.3)	0.6(-0.8,2)	1.5(-1.6,4.5)	-0.6(-2,0.9)	-0.9(-4.3,2.4)
2	-0.9(-2.4,0.7)	-3.2(-9.3,2.9)	0.1(-1.6,1.7)	-0.1(-6.2,6)	0.4(-0.8,1.6)	1.8(-2.4,6.1)	-0.7(-1.9,0.4)	-1.6(-6.1,2.8)
3	-0.6(-1.8,0.6)	-3.9(-11.1,3.3)	0.2(-1.1,1.4)	0(-7.3,7.3)	0.2(-0.8,1.2)	2(-3.1,7.2)	-0.9(-1.8,0.03)	-2.5(-7.9,2.8)
4	-0.3(-1.3,0.6)	-4.3(-12.2,3.6)	0.3(-0.7,1.3)	0.3(-7.9,8.4)	0(-0.8,0.9)	2(-3.9,7.9)	-1(-1.8,-0.3)	-3.6(-9.5,2.4)
5	-0.1(-1,0.7)	-4.6(-12.9,3.8)	0.5(-0.4,1.3)	0.6(-8.1,9.4)	-0.1(-0.9,0.7)	1.9(-4.6,8.3)	-1.2(-1.9,-0.4)	-4.7(-11.1,1.7)
6	0(-0.9,0.8)	-4.5(-13,4)	0.6(-0.3,1.4)	1.1(-8,10.2)	-0.3(-1.1,0.6)	1.5(-5.5,8.5)	-1.2(-2.1,-0.3)	-5.9(-12.7,0.8)
7	0.1(-0.9,1)	-4.1(-12.8,4.5)	0.6(-0.3,1.5)	1.7(-7.6,10.9)	-0.4(-1.3,0.4)	1.1(-6.4,8.5)	-1.2(-2.2,-0.2)	-7.1(-14.1,-0.1)
8	0.2(-0.8,1.2)	-3.6(-12.4,5.2)	0.6(-0.3,1.6)	2.3(-7,11.7)	-0.5(-1.4,0.3)	0.5(-7.4,8.4)	-1.1(-2.1,-0.1)	-8.2(-15.5,-0.9)
9	0.4(-0.6,1.4)	-2.8(-11.8,6.1)	0.7(-0.3,1.7)	3.1(-6.3,12.6)	-0.7(-1.5,0.2)	-0.2(-8.5,8.2)	-0.9(-1.9,0.1)	-9.1(-16.6,-1.6)
10	0.6(-0.4,1.5)	-2(-11.1,7.1)	0.7(-0.2,1.7)	4(-5.5,13.5)	-0.7(-1.6,0.1)	-0.9(-9.7,7.9)	-0.6(-1.5,0.3)	-9.7(-17.5,-1.9)
11	0.8(-0.1,1.6)	-1(-10.3,8.3)	0.8(-0.1,1.7)	4.9(-4.7,14.5)	-0.8(-1.6,-0.01)	-1.7(-11,7.5)	-0.3(-1.1,0.6)	-10(-18,-1.9)
12	0.9(0.2,1.6)	0(-9.5,9.5)	0.8(0.01,1.7)	5.9(-3.8,15.6)	-0.9(-1.6,-0.1)	-2.6(-12.3,7)	0.1(-0.5,0.8)	-9.8(-18.2,-1.5)
13	1(0.3,1.7)	1.1(-8.5,10.7)	0.8(-0.01,1.6)	7(-2.9,16.8)	-0.9(-1.7,-0.2)	-3.6(-13.6,6.5)	0.5(-0.04,1.1)	-9.3(-17.9,-0.8)
14	1.1(0.3,1.9)	2.2(-7.6,12.1)	0.7(-0.1,1.6)	8.1(-2,18.1)	-1(-1.7,-0.3)	-4.6(-15,5.9)	0.9(0.3,1.5)	-8.4(-17.2,0.3)
15	1.3(0.4,2.3)	3.4(-6.6,13.5)	0.7(-0.3,1.6)	9.1(-1.2,19.4)	-1.1(-1.8,-0.3)	-5.5(-16.4,5.3)	1.3(0.6,1.9)	-7.1(-16.1,1.8)
16	1.5(0.4,2.6)	4.6(-5.8,15)	0.6(-0.4,1.7)	10.1(-0.6,20.9)	-1.1(-1.9,-0.3)	-6.5(-17.7,4.7)	1.6(0.9,2.3)	-5.5(-14.6,3.7)
17	1.7(0.5,2.8)	5.9(-4.9,16.7)	0.6(-0.5,1.7)	11.1(-0.3,22.5)	-1.1(-1.9,-0.2)	-7.5(-19.1,4.1)	1.9(1.1,2.7)	-3.6(-13,5.8)
18	1.8(0.6,3)	7.3(-4.1,18.7)	0.6(-0.5,1.7)	11.9(-0.3,24.1)	-1(-1.9,-0.2)	-8.5(-20.5,3.6)	2(1.2,2.9)	-1.5(-11.1,8.2)
19	1.9(0.8,3)	8.9(-3.1,21)	0.6(-0.4,1.7)	12.7(-0.3,25.7)	-1(-1.8,-0.1)	-9.3(-21.9,3.2)	2.1(1.3,2.9)	0.7(-9.2,10.7)
20	2(0.9,3)	10.8(-1.9,23.5)	0.7(-0.3,1.7)	13.5(-0.3,27.3)	-0.9(-1.7,-0.1)	-10.1(-23.1,2.9)	2.2(1.4,2.9)	3(-7.2,13.2)
21	2(1,3.1)	12.9(-0.6,26.3)	0.7(-0.3,1.7)	14.2(-0.3,28.7)	-0.8(-1.6,0.01)	-10.8(-24.2,2.6)	2.1(1.4,2.8)	5.2(-5.3,15.6)
22	2.2(1.1,3.3)	15.2(1,29.4)	0.6(-0.4,1.6)	14.9(-0.2,30)	-0.7(-1.5,0.2)	-11.4(-25.2,2.5)	1.9(1.2,2.7)	7.3(-3.4,17.9)
23	2.3(1,3.7)	17.8(2.9,32.6)	0.6(-0.4,1.7)	15.7(0.1,31.2)	-0.6(-1.6,0.3)	-11.7(-26,2.5)	1.7(0.8,2.6)	9.2(-1.7,20)
24	2.5(0.9,4.1)	20.6(4.9,36.2)	0.7(-0.5,2)	16.4(0.4,32.4)	-0.6(-1.6,0.5)	-12(-26.8,2.8)	1.5(0.3,2.6)	10.9(-0.2,21.9)
25	2.6(0.6,4.6)	23.7(7.1,40.2)	0.8(-0.8,2.3)	17.1(0.7,33.5)	-0.4(-1.7,0.8)	-12(-27.4,3.4)	1.2(-0.2,2.6)	12.2(0.9,23.6)
26	2.7(0.2,5.1)	27(9.4,44.7)	0.9(-1,2.7)	18(1.1,34.8)	-0.3(-1.8,1.2)	-11.9(-28.1,4.2)	0.9(-0.8,2.7)	13.3(1.4,25.2)
**Multi-factor model [b]**								
0	-0.9(-3.5,1.7)	-0.9(-3.5,1.7)	0.1(-2.5,2.6)	0.1(-2.5,2.6)	0.5(-1.3,2.4)	0.5(-1.3,2.4)	0.7(-1.4,2.7)	0.7(-1.4,2.7)
1	-0.8(-2.9,1.4)	-1.7(-6.5,3.1)	0.2(-1.9,2.3)	0.3(-4.4,4.9)	0.4(-1.2,1.9)	0.9(-2.5,4.3)	0.3(-1.4,2)	1(-2.7,4.7)
2	-0.6(-2.3,1.1)	-2.3(-8.8,4.1)	0.3(-1.4,1.9)	0.5(-5.8,6.8)	0.2(-1.1,1.5)	1.1(-3.6,5.8)	0(-1.4,1.3)	1(-4.1,6)
3	-0.4(-1.7,0.9)	-2.8(-10.5,4.9)	0.3(-1,1.6)	0.8(-6.7,8.4)	0(-1.1,1.1)	1.1(-4.6,6.9)	-0.3(-1.4,0.7)	0.7(-5.4,6.7)
4	-0.2(-1.3,0.8)	-3.2(-11.7,5.4)	0.4(-0.6,1.5)	1.2(-7.2,9.7)	-0.2(-1.1,0.8)	1(-5.7,7.6)	-0.6(-1.5,0.3)	0.1(-6.7,6.8)
5	-0.1(-1,0.8)	-3.3(-12.4,5.8)	0.6(-0.3,1.5)	1.7(-7.4,10.8)	-0.3(-1.2,0.6)	0.6(-6.7,8)	-0.9(-1.7,-0.1)	-0.8(-8.1,6.5)
6	-0.1(-1,0.9)	-3.3(-12.7,6.1)	0.7(-0.2,1.5)	2.2(-7.3,11.7)	-0.4(-1.3,0.4)	0.2(-7.7,8.1)	-1.2(-2.1,-0.3)	-1.8(-9.4,5.9)
7	0(-1,1)	-3(-12.6,6.5)	0.7(-0.3,1.6)	2.8(-6.9,12.5)	-0.6(-1.5,0.4)	-0.4(-8.8,8)	-1.4(-2.4,-0.4)	-2.8(-10.8,5.1)
8	0.1(-1,1.1)	-2.6(-12.3,7)	0.7(-0.3,1.7)	3.4(-6.5,13.3)	-0.6(-1.6,0.3)	-1.2(-10,7.7)	-1.4(-2.5,-0.4)	-4(-12.1,4.2)
9	0.2(-0.9,1.2)	-2.1(-11.8,7.6)	0.7(-0.4,1.7)	4.2(-5.9,14.2)	-0.7(-1.6,0.2)	-2(-11.3,7.3)	-1.3(-2.4,-0.3)	-5.1(-13.4,3.3)
10	0.3(-0.7,1.3)	-1.5(-11.3,8.3)	0.7(-0.3,1.7)	4.9(-5.2,15)	-0.8(-1.7,0.1)	-2.9(-12.6,6.8)	-1.1(-2,-0.1)	-6(-14.6,2.7)
11	0.4(-0.5,1.3)	-0.9(-10.9,9.1)	0.7(-0.2,1.6)	5.7(-4.5,15.9)	-0.8(-1.7,0)	-3.8(-13.9,6.3)	-0.7(-1.6,0.1)	-6.6(-15.5,2.3)
12	0.5(-0.3,1.3)	-0.3(-10.4,9.9)	0.6(-0.2,1.5)	6.6(-3.8,16.9)	-0.9(-1.7,-0.1)	-4.8(-15.3,5.8)	-0.3(-1,0.4)	-6.9(-16,2.2)

13	0.5(-0.3,1.3)	0.4(-9.9,10.7)	0.5(-0.3,1.3)	7.4(-3,17.9)	-0.9(-1.7,-0.1)	-5.7(-16.7,5.2)	0.2(-0.5,0.8)	-6.7(-16,2.6)
14	0.6(-0.3,1.4)	1.1(-9.3,11.6)	0.3(-0.5,1.2)	8.3(-2.3,18.9)	-1(-1.7,-0.2)	-6.7(-18,4.7)	0.6(-0.1,1.2)	-6.1(-15.6,3.4)
15	0.7(-0.4,1.8)	1.8(-8.9,12.4)	0.2(-0.8,1.2)	9.2(-1.6,20)	-1(-1.8,-0.2)	-7.6(-19.3,4.1)	1(0.3,1.7)	-5.1(-14.8,4.6)
16	0.9(-0.3,2.1)	2.4(-8.6,13.4)	0.1(-1,1.2)	10(-1.2,21.2)	-1(-1.9,-0.2)	-8.5(-20.7,3.6)	1.3(0.5,2.1)	-3.7(-13.6,6.2)
17	1(-0.3,2.4)	3.1(-8.5,14.7)	0.1(-1.1,1.2)	10.6(-1.2,22.3)	-1(-1.9,-0.1)	-9.4(-22,3.2)	1.6(0.7,2.5)	-2(-12.2,8.2)
18	1.2(-0.2,2.5)	3.9(-8.3,16.2)	0.1(-1.1,1.2)	11(-1.5,23.5)	-1(-1.9,-0.1)	-10.3(-23.3,2.8)	1.8(0.9,2.7)	-0.1(-10.6,10.4)
19	1.3(0,2.6)	5(-8,18.1)	0.2(-0.9,1.3)	11.3(-1.9,24.6)	-0.9(-1.8,-0.1)	-11(-24.5,2.5)	1.9(1.1,2.8)	1.9(-8.9,12.7)
20	1.5(0.3,2.7)	6.4(-7.5,20.3)	0.3(-0.8,1.3)	11.6(-2.4,25.7)	-0.8(-1.6,0)	-11.7(-25.7,2.2)	2(1.2,2.8)	3.9(-7.2,15)
21	1.7(0.5,2.9)	8.1(-6.7,22.8)	0.4(-0.7,1.4)	11.9(-2.9,26.7)	-0.7(-1.5,0.1)	-12.4(-26.8,2.1)	1.9(1.2,2.7)	5.8(-5.5,17.2)
22	2(0.8,3.1)	10.1(-5.5,25.6)	0.4(-0.7,1.4)	12.3(-3.2,27.8)	-0.7(-1.5,0.2)	-12.9(-27.7,2)	1.8(1,2.6)	7.7(-3.9,19.3)
23	2.3(0.9,3.6)	12.4(-4,28.8)	0.4(-0.7,1.6)	12.7(-3.5,28.9)	-0.7(-1.6,0.3)	-13.2(-28.5,2.1)	1.5(0.6,2.5)	9.4(-2.4,21.2)
24	2.5(0.8,4.1)	15.1(-2.1,32.3)	0.6(-0.7,1.9)	13.2(-3.6,30)	-0.6(-1.7,0.5)	-13.5(-29.2,2.2)	1.3(0.1,2.5)	10.9(-1.2,22.9)
25	2.6(0.6,4.7)	18.3(0.1,36.4)	0.7(-0.9,2.4)	13.7(-3.7,31.2)	-0.5(-1.9,0.8)	-13.7(-29.9,2.5)	1(-0.5,2.5)	12(-0.3,24.3)
26	2.8(0.3,5.3)	21.8(2.5,41.1)	0.9(-1.1,2.9)	14.4(-3.7,32.6)	-0.4(-2,1.2)	-13.8(-30.5,2.9)	0.7(-1.1,2.6)	12.8(0,25.6)

[a]*:* Adjusted for WS, AT, RH, SSD and TP;

[b]*:* Adjusted for WS, AT, RH, SSD, TP, and other air pollutants which not in cross-basis;

Abbreviations: $PM_{2.5}$, particulate matter with an aerodynamic diameter of 2.5 μm or less; CO, carbon monoxide; O_X, the combined oxidant capacity; SO_2, Sulfur dioxide; AT, average temperature; WS, wind speed; RH, relative humidity; SSD, sunshine duration; TP, total precipitation.

Table S6. Changes in the excess risk of pulmonary tuberculosis incidence and their 95% CIs for every 10 μg/m^3 increase in O_X, $PM_{2.5}$ and SO_2, and every 0.1 mg/m^3 increase in CO in elderly subgroup.

lag time	$PM_{2.5}$		CO		O_X		SO_2	
	Specific lag time	Cumulative lag time	Specific lag time	Cumulative lag time	Specific lag time	Cumulative lag time	Specific lag time	Cumulative lag time
**Single-factor model** [a]								
0	-0.9(-3.4,1.7)	-0.9(-3.4,1.7)	-0.5(-2.6,1.6)	-0.5(-2.6,1.6)	0.3(-1.9,2.5)	0.3(-1.9,2.5)	-0.7(-2.8,1.4)	-0.7(-2.8,1.4)
1	-0.9(-3,1.2)	-1.7(-6.4,2.9)	-0.4(-2.1,1.3)	-0.9(-4.7,3)	0.2(-1.7,2.1)	0.5(-3.5,4.6)	-0.9(-2.6,0.8)	-1.6(-5.4,2.2)
2	-0.9(-2.5,0.8)	-2.6(-8.9,3.7)	-0.3(-1.7,1.1)	-1.2(-6.4,4)	0.1(-1.5,1.6)	0.6(-5,6.2)	-1(-2.4,0.3)	-2.6(-7.8,2.5)
3	-0.8(-2.1,0.5)	-3.5(-11,4)	-0.2(-1.4,0.9)	-1.5(-7.7,4.8)	0(-1.3,1.3)	0.5(-6.3,7.4)	-1.2(-2.3,-0.1)	-3.8(-9.9,2.4)
4	-0.8(-1.8,0.3)	-4.3(-12.7,4.1)	-0.2(-1.2,0.8)	-1.6(-8.7,5.4)	-0.2(-1.3,1)	0.4(-7.5,8.3)	-1.3(-2.2,-0.4)	-5.1(-11.9,1.8)
5	-0.7(-1.6,0.3)	-5.1(-14.1,3.9)	-0.2(-1.2,0.8)	-1.6(-9.3,6)	-0.3(-1.3,0.7)	0.1(-8.5,8.8)	-1.4(-2.2,-0.5)	-6.4(-13.8,1)
6	-0.6(-1.6,0.4)	-5.7(-15.1,3.6)	-0.1(-1.2,0.9)	-1.5(-9.6,6.5)	-0.4(-1.4,0.5)	-0.2(-9.5,9.1)	-1.4(-2.2,-0.5)	-7.8(-15.5,0)
7	-0.5(-1.6,0.5)	-6.2(-15.8,3.5)	-0.1(-1.2,1.1)	-1.4(-9.8,7)	-0.6(-1.5,0.4)	-0.6(-10.4,9.3)	-1.3(-2.2,-0.3)	-9.1(-17.1,-1.1)
8	-0.4(-1.5,0.8)	-6.4(-16.3,3.4)	0(-1.1,1.2)	-1.1(-9.9,7.6)	-0.6(-1.6,0.4)	-1(-11.4,9.3)	-1.1(-2.1,-0.1)	-10.3(-18.6,-2)
9	-0.1(-1.2,1)	-6.4(-16.5,3.6)	0.2(-1,1.3)	-0.8(-10,8.4)	-0.7(-1.7,0.3)	-1.6(-12.4,9.3)	-0.8(-1.8,0.2)	-11.2(-19.8,-2.6)
10	0.1(-0.9,1.2)	-6.2(-16.5,4)	0.3(-0.7,1.4)	-0.2(-10.1,9.6)	-0.7(-1.6,0.3)	-2.2(-13.5,9.2)	-0.4(-1.4,0.5)	-11.8(-20.7,-2.9)
11	0.4(-0.5,1.4)	-5.8(-16.2,4.7)	0.5(-0.5,1.6)	0.6(-10,11.1)	-0.7(-1.6,0.2)	-2.8(-14.6,9.1)	0(-0.9,0.8)	-12.1(-21.3,-2.8)
12	0.7(-0.1,1.6)	-5.1(-15.8,5.6)	0.7(-0.2,1.6)	1.6(-9.5,12.8)	-0.6(-1.5,0.2)	-3.4(-15.7,9)	0.4(-0.3,1.1)	-11.8(-21.3,-2.3)
13	1(0.2,1.8)	-4.2(-15.1,6.7)	0.8(-0.1,1.7)	2.9(-8.8,14.6)	-0.6(-1.5,0.2)	-4(-16.8,8.9)	0.8(0.2,1.5)	-11.1(-20.9,-1.4)
14	1.2(0.4,2.1)	-3.1(-14.3,8)	0.9(0.04,1.8)	4.5(-7.7,16.6)	-0.7(-1.5,0.2)	-4.5(-17.9,8.9)	1.2(0.5,1.8)	-10(-20,-0.1)
15	1.5(0.5,2.5)	-1.9(-13.3,9.5)	1.01(-0.01,2)	6.1(-6.4,18.7)	-0.7(-1.6,0.3)	-5(-19,9)	1.4(0.7,2.2)	-8.6(-18.8,1.6)
16	1.7(0.6,2.8)	-0.5(-12.3,11.2)	1.1(-0.01,2.2)	7.9(-5,20.8)	-0.7(-1.7,0.4)	-5.5(-20.1,9)	1.6(0.8,2.5)	-6.9(-17.3,3.5)
17	1.8(0.6,3)	0.9(-11.3,13.2)	1.1(-0.01,2.3)	9.7(-3.6,23)	-0.6(-1.7,0.4)	-6.1(-21.3,9.2)	1.7(0.8,2.6)	-5.1(-15.8,5.5)
18	1.8(0.6,3.1)	2.5(-10.5,15.4)	1.1(0.01,2.3)	11.3(-2.4,25)	-0.7(-1.8,0.5)	-6.6(-22.5,9.3)	1.6(0.7,2.6)	-3.4(-14.4,7.5)
19	1.7(0.5,2.9)	3.9(-9.7,17.6)	1.1(0.03,2.2)	12.8(-1.3,26.9)	-0.7(-1.8,0.4)	-7.2(-23.8,9.4)	1.4(0.5,2.3)	-2(-13.3,9.3)
20	1.4(0.3,2.6)	5.3(-9.2,19.8)	1.1(0.05,2.1)	14.1(-0.5,28.6)	-0.7(-1.7,0.3)	-7.8(-25,9.5)	1(0.1,1.8)	-1(-12.6,10.6)
21	1.1(0,2.3)	6.4(-8.9,21.8)	0.9(-0.03,1.9)	15.1(0,30.1)	-0.8(-1.8,0.2)	-8.4(-26.3,9.4)	0.4(-0.4,1.2)	-0.6(-12.5,11.3)
22	0.9(-0.3,2)	7.3(-8.8,23.4)	0.7(-0.3,1.7)	15.7(0.1,31.3)	-0.9(-1.9,0.1)	-9.2(-27.6,9.3)	-0.3(-1.2,0.6)	-1(-13.1,11.2)
23	0.6(-0.7,2)	7.8(-9.1,24.8)	0.4(-0.7,1.6)	16.1(0,32.2)	-1(-2.1,0.04)	-9.9(-28.8,9)	-1(-2.1,0.1)	-2.1(-14.4,10.3)
24	0.3(-1.4,2)	8.1(-9.8,25.9)	0.3(-1.1,1.7)	16(-0.7,32.7)	-1.2(-2.4,0.1)	-10.8(-30.1,8.6)	-1.7(-3.1,-0.3)	-4.1(-16.7,8.5)
25	-0.1(-2.2,2)	8(-10.8,26.9)	0.1(-1.7,1.8)	15.4(-2.1,32.9)	-1.3(-2.8,0.2)	-11.7(-31.5,8.2)	-2.5(-4.2,-0.7)	-7.1(-20,5.8)
26	-0.6(-3.2,1.9)	7.8(-12.3,27.9)	-0.1(-2.2,2)	14.2(-4.4,32.8)	-1.4(-3.2,0.4)	-12.7(-33,7.7)	-3.2(-5.3,-1.1)	-11(-24.5,2.5)
**Multi-factor model** [b]								
0	-1.4(-4.3,1.5)	-1.4(-4.3,1.5)	-0.8(-3.1,1.5)	-0.8(-3.1,1.5)	-0.8(-3.2,1.5)	-0.8(-3.2,1.5)	0.3(-2,2.7)	0.3(-2,2.7)
1	-1.4(-3.8,0.9)	-2.9(-8.1,2.4)	-0.7(-2.6,1.1)	-1.5(-5.7,2.6)	-0.9(-2.9,1.1)	-1.7(-6.1,2.6)	0(-2,1.9)	0.3(-4,4.6)
2	-1.4(-3.2,0.5)	-4.2(-11.3,2.8)	-0.6(-2.1,0.9)	-2.2(-7.8,3.4)	-0.9(-2.6,0.8)	-2.7(-8.7,3.4)	-0.4(-2,1.1)	-0.1(-5.9,5.7)
3	-1.3(-2.8,0.1)	-5.6(-14,2.9)	-0.6(-1.8,0.6)	-2.7(-9.5,4)	-1(-2.4,0.5)	-3.7(-11.1,3.8)	-0.8(-2,0.4)	-0.9(-7.8,6.1)
4	-1.2(-2.3,-0.1)	-6.9(-16.3,2.6)	-0.5(-1.6,0.6)	-3.2(-10.8,4.4)	-1(-2.2,0.2)	-4.7(-13.2,3.9)	-1.2(-2.2,-0.2)	-2(-9.7,5.8)
5	-1(-2,0)	-8(-18.1,2)	-0.4(-1.5,0.7)	-3.6(-11.7,4.6)	-1(-2.1,0.1)	-5.7(-15.1,3.8)	-1.5(-2.4,-0.5)	-3.3(-11.7,5)
6	-0.9(-1.9,0.1)	-9(-19.5,1.4)	-0.3(-1.5,0.9)	-3.8(-12.4,4.8)	-1(-2,0.1)	-6.6(-16.9,3.6)	-1.6(-2.5,-0.7)	-4.9(-13.6,3.9)
7	-0.8(-1.9,0.3)	-9.9(-20.5,0.7)	-0.2(-1.5,1)	-4(-13,5)	-0.9(-2,0.1)	-7.6(-18.5,3.3)	-1.6(-2.6,-0.7)	-6.6(-15.6,2.5)
8	-0.6(-1.8,0.6)	-10.5(-21.3,0.2)	-0.1(-1.4,1.2)	-4.1(-13.4,5.3)	-0.9(-1.9,0.1)	-8.5(-20,2.9)	-1.5(-2.6,-0.5)	-8.2(-17.5,1.1)
9	-0.4(-1.6,0.9)	-10.9(-21.8,0)	0.1(-1.2,1.3)	-3.8(-14,6.3)	-0.8(-1.8,0.2)	-9.4(-21.3,2.6)	-1.2(-2.3,-0.2)	-9.8(-19.3,-0.2)
10	-0.1(-1.3,1.1)	-11.1(-22.1,0)	0.3(-0.9,1.4)	-3.3(-14.3,7.7)	-0.7(-1.7,0.3)	-10.1(-22.6,2.3)	-0.9(-1.9,0.1)	-11(-20.8,-1.2)
11	0.1(-1,1.3)	-11(-22.2,0.2)	0.5(-0.6,1.5)	-2.5(-14.2,9.3)	-0.6(-1.5,0.3)	-10.8(-23.7,2.1)	-0.4(-1.3,0.5)	-11.8(-21.9,-1.7)
12	0.4(-0.7,1.4)	-10.7(-22.1,0.6)	0.6(-0.4,1.6)	-1.4(-13.9,11)	-0.5(-1.3,0.4)	-11.4(-24.7,2)	0.1(-0.7,0.9)	-12.1(-22.4,-1.7)

13	0.5(-0.5,1.5)	-10.3(-21.8,1.3)	0.6(-0.2,1.5)	-0.1(-13.2,12.9)	-0.4(-1.3,0.4)	-11.9(-25.7,1.9)	0.6(-0.2,1.3)	-11.8(-22.4,-1.2)
14	0.7(-0.3,1.8)	-9.6(-21.4,2.1)	0.7(-0.2,1.7)	1.3(-12.2,14.8)	-0.4(-1.3,0.5)	-12.3(-26.5,2)	1(0.2,1.7)	-11(-21.8,-0.2)
15	0.9(-0.2,2.1)	-8.8(-20.9,3.2)	0.8(-0.3,1.8)	2.9(-11,16.8)	-0.3(-1.4,0.7)	-12.6(-27.3,2.2)	1.3(0.5,2.1)	-9.8(-20.8,1.2)
16	1.1(-0.2,2.4)	-7.9(-20.5,4.6)	0.9(-0.3,2)	4.6(-9.7,18.8)	-0.3(-1.5,0.8)	-12.8(-28.1,2.6)	1.5(0.6,2.5)	-8.2(-19.4,3.1)
17	1.2(-0.2,2.6)	-6.9(-20.1,6.2)	0.9(-0.3,2.2)	6.1(-8.5,20.7)	-0.4(-1.5,0.8)	-13(-29,3)	1.7(0.7,2.7)	-6.4(-17.9,5.2)
18	1.2(-0.3,2.6)	-5.9(-19.8,8.1)	1(-0.3,2.2)	7.5(-7.4,22.4)	-0.4(-1.6,0.8)	-13.3(-29.9,3.4)	1.6(0.6,2.7)	-4.5(-16.4,7.3)
19	1.1(-0.4,2.5)	-4.9(-19.7,10)	1(-0.2,2.2)	8.7(-6.5,24)	-0.5(-1.7,0.6)	-13.6(-31,3.8)	1.4(0.4,2.4)	-3(-15.2,9.2)
20	0.9(-0.5,2.2)	-4(-19.8,11.8)	0.9(-0.2,2.1)	9.7(-5.9,25.4)	-0.7(-1.8,0.4)	-14.1(-32.2,4)	0.9(0,1.9)	-1.8(-14.4,10.7)
21	0.6(-0.7,1.9)	-3.3(-20.1,13.5)	0.8(-0.3,1.9)	10.5(-5.5,26.6)	-0.9(-1.9,0.2)	-14.7(-33.5,4.1)	0.3(-0.5,1.2)	-1.3(-14.2,11.6)
22	0.4(-0.9,1.8)	-2.9(-20.6,14.9)	0.5(-0.7,1.7)	11.1(-5.5,27.7)	-1.1(-2.2,0)	-15.6(-35,3.9)	-0.4(-1.3,0.6)	-1.6(-14.7,11.6)
23	0.2(-1.3,1.7)	-2.7(-21.4,16)	0.3(-1.1,1.7)	11.3(-5.9,28.5)	-1.3(-2.5,-0.2)	-16.6(-36.7,3.5)	-1.1(-2.2,0)	-2.7(-16.1,10.6)
24	-0.1(-2,1.7)	-2.8(-22.4,16.9)	0.2(-1.5,1.8)	11.1(-6.9,29.2)	-1.6(-2.9,-0.3)	-17.9(-38.6,2.9)	-1.8(-3.2,-0.5)	-4.9(-18.5,8.7)
25	-0.5(-2.8,1.8)	-3(-23.8,17.7)	0.1(-1.9,2)	10.5(-8.6,29.5)	-1.9(-3.4,-0.3)	-19.3(-40.7,2.1)	-2.7(-4.4,-0.9)	-8.2(-22.1,5.7)
26	-0.9(-3.6,1.9)	-3.5(-25.5,18.5)	0(-2.4,2.3)	9.4(-11.1,29.8)	-2.1(-4,-0.3)	-21(-43.2,1.2)	-3.5(-5.7,-1.3)	-12.6(-27.1,1.9)

[a]*:* Adjusted for WS, AT, RH, SSD and TP;

[b]*:* Adjusted for WS, AT, RH, SSD, TP, and other air pollutants which not in cross-basis;

Abbreviations: $PM_{2.5}$, particulate matter with an aerodynamic diameter of 2.5 μm or less; CO, carbon monoxide; O_X, the combined oxidant capacity; SO_2, Sulfur dioxide; AT, average temperature; WS, wind speed; RH, relative humidity; SSD, sunshine duration; TP, total precipitation.

Table S7. Changes in the excess risk of pulmonary tuberculosis incidence and their 95% CIs for every 10 μg/m^3 increase in O_X, $PM_{2.5}$ and SO_2, and every 0.1 mg/m^3 increase in CO in lower NDVI areas.

lag time	$PM_{2.5}$		CO		O_X		SO_2	
	Specific lag time	Cumulative lag time	Specific lag time	Cumulative lag time	Specific lag time	Cumulative lag time	Specific lag time	Cumulative lag time
**Single-factor model [a]**								
0	-2.1(-4.6,0.4)	-2.1(-4.6,0.4)	0.7(-1.6,3)	0.7(-1.6,3)	2(0.1,3.9)	2(0.1,3.9)	-0.2(-2.3,1.8)	-0.2(-2.3,1.8)
1	-1.7(-3.7,0.3)	-3.9(-8.4,0.7)	0.6(-1.4,2.5)	1.3(-3,5.5)	1.7(-0.01,3.3)	3.7(0.1,7.3)	-0.5(-2.1,1.1)	-0.7(-4.4,2.9)
2	-1.3(-2.9,0.3)	-5.2(-11.3,0.9)	0.4(-1.1,1.9)	1.7(-4.1,7.4)	1.3(-0.1,2.7)	5(-0.03,10)	-0.8(-2,0.5)	-1.5(-6.4,3.4)
3	-0.9(-2.1,0.3)	-6.1(-13.3,1.1)	0.3(-0.9,1.5)	2(-4.9,8.8)	1(-0.3,2.2)	5.9(-0.3,12.1)	-1(-2,0.03)	-2.5(-8.3,3.3)
4	-0.4(-1.3,0.5)	-6.6(-14.6,1.4)	0.2(-0.8,1.1)	2.1(-5.6,9.8)	0.7(-0.4,1.8)	6.5(-0.7,13.7)	-1.2(-2,-0.3)	-3.6(-10.1,2.8)
5	0(-0.9,0.8)	-6.7(-15.2,1.7)	0.1(-0.8,1)	2.2(-6.1,10.5)	0.4(-0.6,1.4)	6.9(-1.2,15)	-1.3(-2.1,-0.5)	-4.9(-11.9,2)
6	0.2(-0.7,1.1)	-6.4(-15.1,2.3)	0.1(-0.8,1)	2.2(-6.5,10.9)	0.2(-0.8,1.2)	7(-1.8,15.8)	-1.4(-2.3,-0.4)	-6.4(-13.7,1)
7	0.4(-0.7,1.5)	-5.9(-14.7,3)	0.1(-0.9,1.1)	2.2(-6.7,11.2)	-0.1(-1,0.9)	6.9(-2.7,16.4)	-1.3(-2.4,-0.3)	-7.8(-15.4,-0.1)
8	0.5(-0.6,1.7)	-5.1(-14,3.9)	0.1(-1,1.2)	2.3(-6.9,11.4)	-0.3(-1.3,0.7)	6.6(-3.6,16.7)	-1.2(-2.3,-0.1)	-9.1(-17,-1.2)
9	0.6(-0.5,1.8)	-4.1(-13.1,4.9)	0.2(-0.9,1.3)	2.4(-7,11.8)	-0.5(-1.4,0.5)	6.1(-4.7,16.9)	-1(-2.1,0.1)	-10.2(-18.3,-2)
10	0.7(-0.3,1.7)	-3.2(-12.3,6)	0.3(-0.8,1.4)	2.7(-7,12.4)	-0.6(-1.6,0.4)	5.6(-5.9,17)	-0.7(-1.7,0.3)	-10.9(-19.4,-2.5)
11	0.7(-0.1,1.6)	-2.3(-11.6,7)	0.4(-0.6,1.5)	3.2(-6.8,13.2)	-0.7(-1.6,0.3)	4.9(-7.1,16.9)	-0.3(-1.2,0.6)	-11.3(-20,-2.5)
12	0.7(0,1.4)	-1.6(-11,7.8)	0.6(-0.4,1.6)	3.9(-6.4,14.3)	-0.7(-1.7,0.2)	4.2(-8.5,16.8)	0.2(-0.6,0.9)	-11.2(-20.2,-2.2)
13	0.6(-0.1,1.2)	-1(-10.6,8.6)	0.7(-0.4,1.7)	4.9(-5.8,15.7)	-0.8(-1.7,0.1)	3.4(-9.9,16.7)	0.6(-0.05,1.3)	-10.7(-19.9,-1.4)
14	0.5(-0.3,1.2)	-0.6(-10.3,9.1)	0.7(-0.3,1.8)	6.2(-5,17.4)	-0.8(-1.6,0)	2.6(-11.3,16.5)	1(0.3,1.7)	-9.7(-19.2,-0.3)
15	0.5(-0.5,1.5)	-0.3(-10.2,9.6)	0.8(-0.4,2)	7.6(-4.1,19.4)	-0.8(-1.7,0.1)	1.8(-12.7,16.3)	1.4(0.6,2.1)	-8.4(-18.1,1.3)
16	0.6(-0.6,1.8)	-0.1(-10.3,10.1)	0.9(-0.4,2.1)	9.1(-3.3,21.5)	-0.8(-1.7,0.1)	1.1(-14,16.1)	1.6(0.8,2.5)	-6.7(-16.6,3.1)
17	0.7(-0.7,2)	0.1(-10.5,10.8)	0.9(-0.4,2.2)	10.5(-2.7,23.8)	-0.7(-1.6,0.2)	0.4(-15.2,16)	1.8(0.8,2.7)	-4.9(-15,5.2)
18	0.8(-0.6,2.2)	0.5(-10.7,11.7)	1(-0.3,2.2)	11.8(-2.3,25.9)	-0.6(-1.5,0.3)	-0.1(-16.2,16)	1.8(0.8,2.8)	-3(-13.4,7.4)
19	1(-0.4,2.3)	1.1(-10.8,13)	1(-0.2,2.2)	12.9(-2,27.9)	-0.5(-1.4,0.4)	-0.5(-17.1,16.1)	1.7(0.7,2.6)	-1.2(-11.9,9.5)
20	1.2(-0.1,2.4)	2.1(-10.6,14.7)	1(-0.1,2.1)	14(-1.7,29.7)	-0.3(-1.2,0.5)	-0.7(-17.7,16.3)	1.4(0.6,2.3)	0.3(-10.7,11.4)
21	1.4(0.3,2.6)	3.4(-10,16.8)	1(-0.02,2.1)	15(-1.4,31.3)	-0.2(-1.1,0.7)	-0.7(-18.1,16.7)	1.1(0.3,1.9)	1.4(-9.8,12.7)
22	1.8(0.6,2.9)	5.2(-8.9,19.3)	1(0.03,2)	16(-0.8,32.8)	-0.1(-1,0.8)	-0.5(-18.2,17.2)	0.6(-0.2,1.5)	2(-9.5,13.6)
23	2.3(1,3.5)	7.5(-7.3,22.3)	1(-0.2,2.1)	16.9(-0.2,34)	0.1(-0.9,1)	-0.1(-18.1,17.9)	0.1(-0.8,1.1)	2(-9.7,13.8)
24	2.8(1.2,4.3)	10.3(-5.3,25.8)	0.9(-0.4,2.2)	17.9(0.6,35.1)	0.2(-0.9,1.3)	0.6(-17.7,18.9)	-0.4(-1.7,0.8)	1.3(-10.7,13.3)
25	3.2(1.3,5.2)	13.7(-2.7,30.1)	0.9(-0.8,2.5)	18.8(1.6,36)	0.4(-1,1.7)	1.5(-17.1,20.1)	-1(-2.7,0.6)	-0.1(-12.4,12.3)
26	3.7(1.2,6.2)	17.7(0.2,35.2)	0.9(-1.1,2.9)	19.3(1.3,37.3)	0.5(-1.1,2.1)	2.6(-16.4,21.6)	-1.7(-3.7,0.4)	-2.2(-15.1,10.7)
**Multi-factor model [b]**								
0	-1.8(-4.6,1.1)	-1.8(-4.6,1.1)	1(-1.3,3.3)	1(-1.3,3.3)	2.1(0.2,4)	2.1(0.2,4)	0.5(-1.6,2.7)	0.5(-1.6,2.7)
1	-1.5(-3.7,0.8)	-3.2(-8.3,1.9)	0.8(-1.2,2.7)	1.7(-2.5,5.9)	1.7(0.1,3.4)	3.8(0.2,7.4)	0.1(-1.6,1.9)	0.7(-3.3,4.7)
2	-1.1(-2.9,0.6)	-4.4(-11.2,2.5)	0.5(-1,2.1)	2.3(-3.5,8)	1.4(-0.1,2.8)	5.2(0.2,10.2)	-0.2(-1.7,1.2)	0.5(-4.9,5.9)
3	-0.8(-2.2,0.5)	-5.2(-13.4,2.9)	0.3(-0.9,1.5)	2.6(-4.3,9.4)	1(-0.3,2.3)	6.2(-0.1,12.5)	-0.6(-1.8,0.5)	-0.1(-6.6,6.4)
4	-0.5(-1.5,0.5)	-5.8(-14.8,3.2)	0.1(-0.9,1.1)	2.7(-5,10.4)	0.7(-0.5,1.8)	6.9(-0.4,14.2)	-1(-1.9,0)	-1(-8.3,6.2)
5	-0.2(-1.1,0.7)	-6.1(-15.6,3.4)	0(-0.9,0.9)	2.7(-5.6,11)	0.4(-0.7,1.5)	7.2(-1,15.4)	-1.3(-2.1,-0.4)	-2.2(-10,5.6)
6	0(-1,0.9)	-6.1(-15.8,3.6)	-0.1(-1.1,0.9)	2.5(-6.2,11.2)	0.2(-0.9,1.2)	7.3(-1.7,16.3)	-1.5(-2.5,-0.6)	-3.6(-11.8,4.6)
7	0.1(-1.1,1.2)	-5.9(-15.7,3.9)	-0.2(-1.2,0.9)	2.2(-6.8,11.2)	-0.1(-1.2,1)	7.2(-2.6,16.9)	-1.6(-2.7,-0.6)	-5.1(-13.6,3.4)
8	0.1(-1.1,1.4)	-5.5(-15.3,4.2)	-0.2(-1.4,1)	1.9(-7.4,11.2)	-0.3(-1.4,0.8)	6.8(-3.6,17.3)	-1.6(-2.7,-0.5)	-6.7(-15.4,2.1)
9	0.2(-1,1.4)	-5.1(-14.8,4.7)	-0.2(-1.4,1)	1.7(-8,11.3)	-0.4(-1.5,0.7)	6.4(-4.7,17.5)	-1.4(-2.5,-0.3)	-8.1(-17.1,0.9)
10	0.2(-0.9,1.3)	-4.6(-14.4,5.2)	-0.1(-1.3,1.1)	1.6(-8.4,11.6)	-0.5(-1.6,0.6)	5.8(-5.9,17.5)	-1(-2.1,0)	-9.2(-18.5,0)
11	0.2(-0.8,1.1)	-4.3(-14.1,5.6)	0(-1.1,1.2)	1.7(-8.7,12.1)	-0.6(-1.7,0.5)	5.2(-7.1,17.6)	-0.6(-1.5,0.3)	-10(-19.5,-0.5)
12	0.1(-0.7,0.9)	-4(-14,6)	0.1(-1,1.2)	2(-8.8,12.9)	-0.6(-1.6,0.4)	4.6(-8.4,17.6)	-0.1(-0.9,0.7)	-10.3(-20,-0.5)

13	0(-0.8,0.8)	-3.9(-14,6.2)	0.2(-0.9,1.3)	2.7(-8.7,14)	-0.6(-1.6,0.3)	3.9(-9.7,17.5)	0.4(-0.3,1.1)	-10(-20,-0.1)
14	-0.1(-1,0.8)	-3.9(-14.2,6.3)	0.2(-0.9,1.3)	3.5(-8.4,15.5)	-0.6(-1.5,0.2)	3.3(-10.9,17.5)	0.9(0.2,1.6)	-9.3(-19.5,0.9)
15	-0.1(-1.1,0.9)	-4.1(-14.6,6.3)	0.3(-0.9,1.5)	4.5(-8.1,17.2)	-0.6(-1.5,0.3)	2.7(-12.1,17.5)	1.3(0.5,2.2)	-8.1(-18.5,2.3)
16	0(-1.2,1.2)	-4.4(-15.2,6.4)	0.3(-0.9,1.6)	5.5(-7.9,19)	-0.6(-1.5,0.3)	2.1(-13.2,17.5)	1.6(0.7,2.6)	-6.5(-17.2,4.1)
17	0.1(-1.2,1.4)	-4.7(-16,6.7)	0.4(-0.9,1.7)	6.5(-7.8,20.7)	-0.6(-1.5,0.4)	1.7(-14.3,17.6)	1.8(0.8,2.9)	-4.7(-15.6,6.3)
18	0.3(-1.1,1.6)	-4.8(-16.9,7.3)	0.5(-0.8,1.8)	7.3(-7.8,22.4)	-0.5(-1.5,0.5)	1.3(-15.2,17.7)	1.9(0.9,2.9)	-2.7(-14,8.5)
19	0.5(-0.8,1.8)	-4.6(-17.5,8.3)	0.6(-0.7,1.9)	8(-7.9,23.9)	-0.4(-1.3,0.6)	1(-15.9,17.9)	1.8(0.8,2.7)	-0.9(-12.5,10.7)
20	0.8(-0.5,2)	-4(-17.8,9.8)	0.7(-0.5,1.9)	8.7(-8,25.4)	-0.3(-1.2,0.6)	0.8(-16.5,18.1)	1.5(0.7,2.4)	0.7(-11.2,12.6)
21	1.1(-0.1,2.3)	-3(-17.6,11.7)	0.8(-0.4,1.9)	9.4(-7.8,26.7)	-0.2(-1.1,0.7)	0.8(-16.8,18.4)	1.2(0.4,2)	1.9(-10.3,14.1)
22	1.5(0.4,2.7)	-1.4(-16.9,14.1)	0.9(-0.2,2)	10.2(-7.5,27.9)	-0.2(-1.1,0.8)	0.9(-17,18.8)	0.8(-0.1,1.6)	2.6(-9.9,15.1)
23	2.1(0.8,3.4)	0.7(-15.6,17)	0.9(-0.3,2.1)	11.1(-6.9,29.1)	-0.1(-1.1,0.9)	1.1(-17,19.3)	0.2(-0.8,1.3)	2.7(-10.1,15.4)
24	2.6(1,4.2)	3.4(-13.7,20.5)	0.9(-0.5,2.4)	12.1(-6,30.2)	-0.1(-1.3,1.1)	1.5(-16.9,19.9)	-0.3(-1.6,0.9)	2(-10.9,15)
25	3.1(1.2,5.1)	6.8(-11.2,24.8)	1(-0.7,2.7)	13.3(-5,31.5)	0(-1.4,1.4)	2(-16.8,20.7)	-1(-2.7,0.7)	0.7(-12.6,14)
26	3.6(1.1,6.1)	10.8(-8.2,29.9)	1.1(-1,3.2)	14(-5,33.1)	0.1(-1.6,1.7)	2.5(-16.7,21.8)	-1.7(-3.7,0.4)	-1.4(-15.2,12.4)

^{a}: Adjusted for WS, AT, RH, SSD and TP;

^{b}: Adjusted for WS, AT, RH, SSD, TP, and other air pollutants which not in cross-basis;

Abbreviations: $PM_{2.5}$, particulate matter with an aerodynamic diameter of 2.5 μm or less; CO, carbon monoxide; O_X, the combined oxidant capacity; SO_2, Sulfur dioxide; AT, average temperature; WS, wind speed; RH, relative humidity; SSD, sunshine duration; TP, total precipitation.

Table S8. Changes in the excess risk of pulmonary tuberculosis incidence and their 95% CIs for every 10 μg/m^3 increase in O_X, $PM_{2.5}$ and SO_2, and every 0.1 mg/m^3 increase in CO in higher NDVI areas.

lag time	$PM_{2.5}$		CO		O_X		SO_2	
	Specific lag time	Cumulative lag time	Specific lag time	Cumulative lag time	Specific lag time	Cumulative lag time	Specific lag time	Cumulative lag time
**Single-factor model [a]**								
0	-0.6(-3.1,1.9)	-0.6(-3.1,1.9)	-1.5(-3.9,1)	-1.5(-3.9,1)	-0.7(-2.6,1.1)	-0.7(-2.6,1.1)	-0.7(-2.7,1.4)	-0.7(-2.7,1.4)
1	-0.7(-2.8,1.4)	-1.3(-5.9,3.2)	-1.1(-3.1,0.9)	-2.6(-7,1.8)	-0.7(-2.3,0.8)	-1.5(-4.9,2)	-0.8(-2.5,0.8)	-1.5(-5.2,2.2)
2	-0.8(-2.4,0.9)	-2.1(-8.3,4.1)	-0.8(-2.3,0.8)	-3.3(-9.3,2.6)	-0.7(-2,0.6)	-2.2(-6.9,2.5)	-0.9(-2.3,0.4)	-2.4(-7.4,2.5)
3	-0.8(-2.2,0.5)	-2.9(-10.4,4.6)	-0.4(-1.7,0.8)	-3.8(-10.9,3.4)	-0.7(-1.8,0.4)	-2.9(-8.6,2.8)	-1.1(-2.1,-0.01)	-3.5(-9.4,2.5)
4	-0.9(-2,0.2)	-3.7(-12.2,4.7)	-0.1(-1.2,0.9)	-3.8(-11.9,4.2)	-0.7(-1.7,0.2)	-3.6(-10.1,3)	-1.1(-2,-0.3)	-4.6(-11.2,2)
5	-0.9(-1.9,0.1)	-4.5(-13.7,4.6)	0.1(-0.9,1)	-3.6(-12.3,5)	-0.8(-1.7,0.1)	-4.2(-11.4,2.9)	-1.2(-2,-0.3)	-5.8(-12.9,1.3)
6	-0.9(-1.9,0.1)	-5.3(-14.9,4.3)	0.3(-0.7,1.3)	-3.2(-12.2,5.8)	-0.8(-1.7,0.1)	-4.9(-12.6,2.7)	-1.2(-2,-0.3)	-6.9(-14.4,0.5)
7	-0.8(-1.9,0.3)	-6(-16,4)	0.4(-0.6,1.5)	-2.6(-11.9,6.8)	-0.8(-1.8,0.1)	-5.7(-13.8,2.4)	-1.1(-2,-0.2)	-8(-15.8,-0.3)
8	-0.6(-1.9,0.6)	-6.5(-16.8,3.8)	0.6(-0.5,1.7)	-1.8(-11.4,7.8)	-0.8(-1.8,0.2)	-6.5(-15,2.1)	-1(-1.9,0)	-8.9(-17,-0.9)
9	-0.4(-1.6,0.9)	-6.8(-17.3,3.8)	0.7(-0.4,1.8)	-1(-10.9,9)	-0.8(-1.8,0.1)	-7.3(-16.3,1.7)	-0.7(-1.7,0.2)	-9.7(-18,-1.4)
10	0(-1.2,1.2)	-6.7(-17.5,4.2)	0.8(-0.3,1.9)	0(-10.3,10.2)	-0.8(-1.7,0.1)	-8.2(-17.6,1.2)	-0.4(-1.3,0.5)	-10.1(-18.7,-1.5)
11	0.5(-0.7,1.6)	-6.1(-17.3,5)	0.8(-0.2,1.8)	0.9(-9.7,11.5)	-0.8(-1.6,0.1)	-9.1(-19,0.8)	0(-0.8,0.8)	-10.1(-19.1,-1.2)
12	0.9(-0.1,1.9)	-5.2(-16.6,6.3)	0.8(-0.1,1.7)	2(-9,12.9)	-0.7(-1.6,0.1)	-10(-20.3,0.3)	0.4(-0.3,1.1)	-9.8(-19,-0.5)
13	1.4(0.5,2.3)	-3.7(-15.4,8.1)	0.7(-0.1,1.5)	3(-8.2,14.3)	-0.8(-1.6,0.1)	-10.8(-21.5,-0.1)	0.9(0.2,1.5)	-9(-18.4,0.5)
14	1.8(0.9,2.7)	-1.8(-13.8,10.3)	0.6(-0.2,1.4)	4.1(-7.4,15.7)	-0.8(-1.7,-0.01)	-11.7(-22.8,-0.6)	1.2(0.6,1.9)	-7.8(-17.5,1.9)
15	2.1(1.1,3.1)	0.5(-11.9,12.9)	0.6(-0.3,1.5)	5.2(-6.6,17)	-0.9(-1.8,-0.04)	-12.6(-24.1,-1.1)	1.5(0.8,2.3)	-6.3(-16.1,3.6)
16	2.4(1.3,3.5)	3.1(-9.7,15.9)	0.6(-0.4,1.6)	6.2(-5.9,18.4)	-1(-1.9,-0.05)	-13.5(-25.4,-1.6)	1.8(1,2.7)	-4.4(-14.5,5.7)
17	2.5(1.3,3.7)	5.8(-7.6,19.2)	0.5(-0.5,1.6)	7.1(-5.5,19.7)	-1(-2,-0.1)	-14.4(-26.7,-2)	2(1.1,2.9)	-2.4(-12.7,7.9)
18	2.5(1.3,3.8)	8.5(-5.6,22.5)	0.5(-0.6,1.6)	7.9(-5.3,21)	-1.1(-2.1,-0.1)	-15.2(-28.2,-2.2)	2.1(1.2,3)	-0.3(-10.9,10.3)
19	2.4(1.2,3.6)	11(-3.8,25.8)	0.5(-0.5,1.6)	8.5(-5.3,22.2)	-1.1(-2.1,-0.1)	-16.2(-29.9,-2.5)	2.1(1.2,3)	1.8(-9.2,12.7)
20	2.1(1,3.3)	13.2(-2.4,28.8)	0.5(-0.4,1.5)	8.9(-5.5,23.4)	-1.1(-2.1,-0.1)	-17.2(-31.6,-2.7)	1.9(1.1,2.8)	3.7(-7.5,15)
21	1.8(0.6,3)	15(-1.4,31.5)	0.3(-0.7,1.4)	9.2(-5.9,24.4)	-1.2(-2.2,-0.1)	-18.3(-33.5,-3)	1.7(0.9,2.5)	5.5(-6,17)
22	1.3(0.1,2.6)	16.4(-0.9,33.7)	0.2(-1,1.3)	9.3(-6.6,25.3)	-1.2(-2.3,-0.1)	-19.4(-35.4,-3.4)	1.4(0.5,2.2)	7(-4.7,18.8)
23	0.8(-0.7,2.3)	17.2(-1,35.4)	0.1(-1.3,1.4)	9.2(-7.6,26)	-1.3(-2.6,-0.1)	-20.6(-37.5,-3.7)	1(0,2)	8.2(-3.7,20.1)
24	0.2(-1.7,2)	17.4(-1.8,36.5)	0.1(-1.5,1.7)	8.9(-8.9,26.6)	-1.5(-2.9,-0.1)	-21.9(-39.7,-4)	0.6(-0.7,1.9)	9(-3.2,21.1)
25	-0.5(-2.8,1.8)	16.9(-3.4,37.2)	0.2(-1.8,2.1)	8.3(-10.6,27.2)	-1.7(-3.3,-0.1)	-23.2(-42.1,-4.4)	0.2(-1.5,1.8)	9.2(-3.5,21.8)
26	-1.1(-3.9,1.6)	15.7(-5.9,37.4)	0.2(-2.1,2.5)	7.6(-12.8,28)	-1.9(-3.6,-0.1)	-24.7(-44.8,-4.7)	-0.3(-2.3,1.7)	8.7(-5,22.3)
**Multi-factor model [b]**								
0	-1.1(-3.9,1.8)	-1.1(-3.9,1.8)	-2(-4.5,0.6)	-2(-4.5,0.6)	-2.4(-4.5,-0.3)	-2.4(-4.5,-0.3)	0.5(-1.8,2.8)	0.5(-1.8,2.8)
1	-1.1(-3.4,1.2)	-2.2(-7.4,3)	-1.5(-3.6,0.6)	-3.5(-8.1,1.1)	-2.2(-4,-0.4)	-4.6(-8.5,-0.7)	0.2(-1.7,2.1)	0.7(-3.5,4.9)
2	-1.1(-3,0.7)	-3.3(-10.3,3.7)	-1.1(-2.7,0.6)	-4.5(-10.8,1.7)	-2(-3.5,-0.5)	-6.6(-11.9,-1.2)	-0.2(-1.7,1.3)	0.5(-5.1,6.2)
3	-1.1(-2.6,0.4)	-4.4(-12.9,4)	-0.6(-2,0.7)	-5.2(-12.7,2.4)	-1.8(-3,-0.6)	-8.4(-14.9,-1.9)	-0.5(-1.7,0.7)	0.1(-6.7,6.8)
4	-1.1(-2.3,0.1)	-5.6(-15,3.9)	-0.3(-1.5,0.9)	-5.4(-13.9,3.1)	-1.7(-2.7,-0.6)	-9.9(-17.3,-2.6)	-0.8(-1.7,0.2)	-0.7(-8.2,6.9)
5	-1(-2.1,0)	-6.6(-16.8,3.6)	0(-1.1,1.1)	-5.3(-14.4,3.9)	-1.5(-2.5,-0.6)	-11.4(-19.5,-3.3)	-1(-1.9,-0.2)	-1.6(-9.7,6.5)
6	-0.9(-2,0.1)	-7.5(-18.2,3.1)	0.3(-0.8,1.5)	-4.9(-14.6,4.8)	-1.4(-2.3,-0.5)	-12.7(-21.3,-4.1)	-1.2(-2.1,-0.3)	-2.7(-11.2,5.8)
7	-0.9(-2.1,0.4)	-8.3(-19.3,2.7)	0.6(-0.6,1.8)	-4.3(-14.4,5.9)	-1.3(-2.2,-0.3)	-13.9(-23,-4.8)	-1.3(-2.3,-0.4)	-3.9(-12.7,4.9)
8	-0.7(-2,0.6)	-8.9(-20.2,2.3)	0.8(-0.4,2)	-3.5(-14.1,7.1)	-1.1(-2.1,-0.1)	-15(-24.5,-5.5)	-1.3(-2.3,-0.3)	-5.1(-14.1,3.9)
9	-0.5(-1.9,0.9)	-9.2(-20.7,2.2)	0.9(-0.2,2.1)	-2.6(-13.6,8.5)	-1(-2,0)	-16.1(-26,-6.2)	-1.2(-2.2,-0.2)	-6.2(-15.5,3.1)
10	-0.2(-1.5,1.1)	-9.2(-21,2.5)	1(-0.1,2.1)	-1.6(-13.1,10)	-0.9(-1.9,0)	-17.1(-27.4,-6.8)	-0.9(-1.8,0)	-7(-16.6,2.5)
11	0.2(-1.1,1.4)	-8.9(-20.8,3.1)	1(0,2)	-0.5(-12.5,11.6)	-0.8(-1.7,0.1)	-18(-28.8,-7.3)	-0.5(-1.4,0.3)	-7.6(-17.4,2.2)
12	0.6(-0.6,1.8)	-8.1(-20.3,4.1)	0.9(0,1.8)	0.7(-11.8,13.2)	-0.7(-1.6,0.2)	-18.9(-30.1,-7.8)	-0.1(-0.8,0.7)	-7.7(-17.8,2.4)

13	1(-0.2,2.1)	-6.9(-19.4,5.6)	0.7(-0.1,1.5)	1.9(-11,14.7)	-0.7(-1.6,0.2)	-19.7(-31.3,-8.2)	0.4(-0.3,1.2)	-7.4(-17.7,2.9)
14	1.3(0.1,2.5)	-5.4(-18.2,7.4)	0.5(-0.3,1.4)	3.1(-10.1,16.2)	-0.7(-1.6,0.2)	-20.5(-32.4,-8.5)	0.8(0.1,1.6)	-6.6(-17.1,3.9)
15	1.6(0.2,2.9)	-3.6(-16.9,9.7)	0.4(-0.5,1.4)	4.2(-9.2,17.6)	-0.7(-1.7,0.3)	-21.2(-33.6,-8.9)	1.2(0.4,2)	-5.3(-16.1,5.4)
16	1.7(0.3,3.2)	-1.6(-15.5,12.3)	0.3(-0.8,1.4)	5.3(-8.3,18.9)	-0.8(-1.8,0.3)	-21.9(-34.7,-9.2)	1.5(0.6,2.4)	-3.7(-14.7,7.2)
17	1.8(0.3,3.3)	0.5(-14.1,15.1)	0.3(-0.9,1.4)	6.2(-7.7,20.1)	-0.8(-2,0.3)	-22.7(-35.9,-9.4)	1.7(0.7,2.7)	-1.9(-13.1,9.3)
18	1.8(0.3,3.3)	2.5(-12.9,17.9)	0.2(-1,1.4)	7(-7.4,21.3)	-0.9(-2,0.3)	-23.3(-37.3,-9.2)	1.8(0.8,2.8)	0.1(-11.4,11.6)
19	1.7(0.2,3.1)	4.3(-12,20.6)	0.2(-0.9,1.4)	7.5(-7.3,22.3)	-0.9(-2.1,0.2)	-23.9(-38.8,-9.1)	1.7(0.8,2.7)	2.1(-9.7,13.9)
20	1.5(0.1,2.9)	5.9(-11.4,23.1)	0.3(-0.8,1.4)	7.9(-7.5,23.3)	-1(-2.1,0.1)	-24.7(-40.4,-9)	1.6(0.7,2.5)	3.9(-8.3,16.1)
21	1.2(-0.2,2.6)	7.1(-11.1,25.3)	0.1(-1,1.3)	8(-8,24.1)	-1.1(-2.2,0)	-25.7(-42.3,-9.1)	1.4(0.5,2.2)	5.5(-7,17.9)
22	0.8(-0.7,2.3)	8(-11.2,27.1)	0(-1.3,1.3)	7.9(-9,24.9)	-1.2(-2.4,-0.1)	-26.8(-44.2,-9.3)	1(0.1,1.9)	6.7(-6,19.4)
23	0.3(-1.3,2)	8.4(-11.7,28.5)	-0.1(-1.6,1.4)	7.6(-10.3,25.6)	-1.4(-2.6,-0.3)	-28(-46.3,-9.7)	0.6(-0.4,1.7)	7.5(-5.4,20.5)
24	-0.2(-2.1,1.8)	8.3(-12.9,29.4)	0(-1.8,1.8)	7.1(-12.1,26.3)	-1.7(-3,-0.4)	-29.4(-48.6,-10.2)	0.2(-1.1,1.5)	7.9(-5.2,21)
25	-0.7(-3.1,1.7)	7.6(-14.8,30)	0.1(-2,2.2)	6.4(-14.2,27)	-2(-3.4,-0.5)	-30.9(-51.1,-10.8)	-0.3(-2,1.4)	7.7(-5.8,21.1)
26	-1.2(-4.1,1.6)	6.3(-17.6,30.3)	0.2(-2.3,2.7)	5.7(-16.5,28)	-2.1(-3.9,-0.3)	-32.8(-54,-11.6)	-0.8(-2.8,1.3)	6.8(-7.2,20.7)

[a]: Adjusted for WS, AT, RH, SSD and TP;

[b]: Adjusted for WS, AT, RH, SSD, TP, and other air pollutants which not in cross-basis;

Abbreviations: $PM_{2.5}$, particulate matter with an aerodynamic diameter of 2.5 μm or less; CO, carbon monoxide; O_X, the combined oxidant capacity; SO_2, Sulfur dioxide; AT, average temperature; WS, wind speed; RH, relative humidity; SSD, sunshine duration; TP, total precipitation.

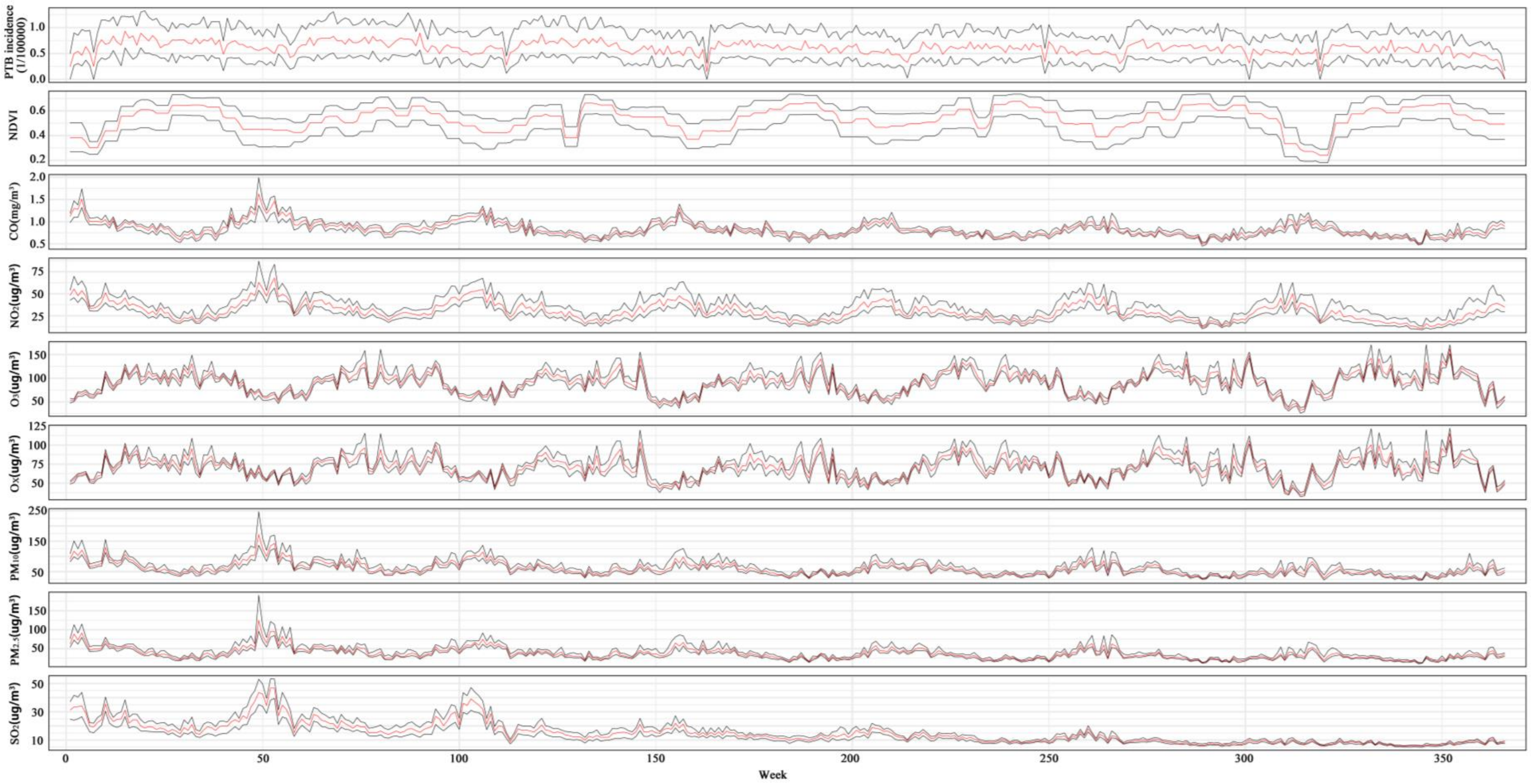

Figure S1. Time series of daily pulmonary tuberculosis incidence and air pollutant level averaged from 2013 to 2019. The red lines represented the median of daily PTB incidence and concentrations of air pollutants and the black lines (top and bottom) represented the IQR (25%, 75%).

Abbreviations: CO, carbon monoxide; NO_2, nitrogen dioxide; O_3, ozone; O_X, the combined oxidant capacity; PM, particulate matter; SO_2, Sulfur dioxide.

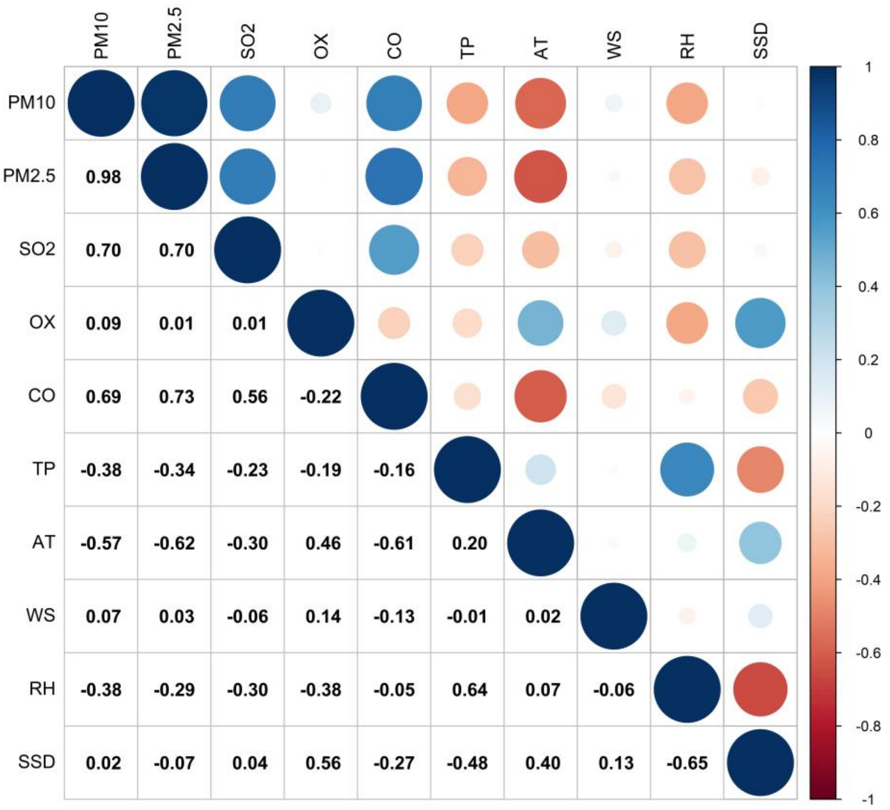

Figure S2. Spearman rank correlation coefficients among air pollutant concentrations and meteorological data.

Abbreviations: $PM_{2.5}$, particulate matter with an aerodynamic diameter of 2.5 μm or less; CO, carbon monoxide; O_X, the combined oxidant capacity; SO_2, Sulfur dioxide; AT, average temperature; WS, wind speed; RH, relative humidity; SSD, sunshine duration; TP, total precipitation.

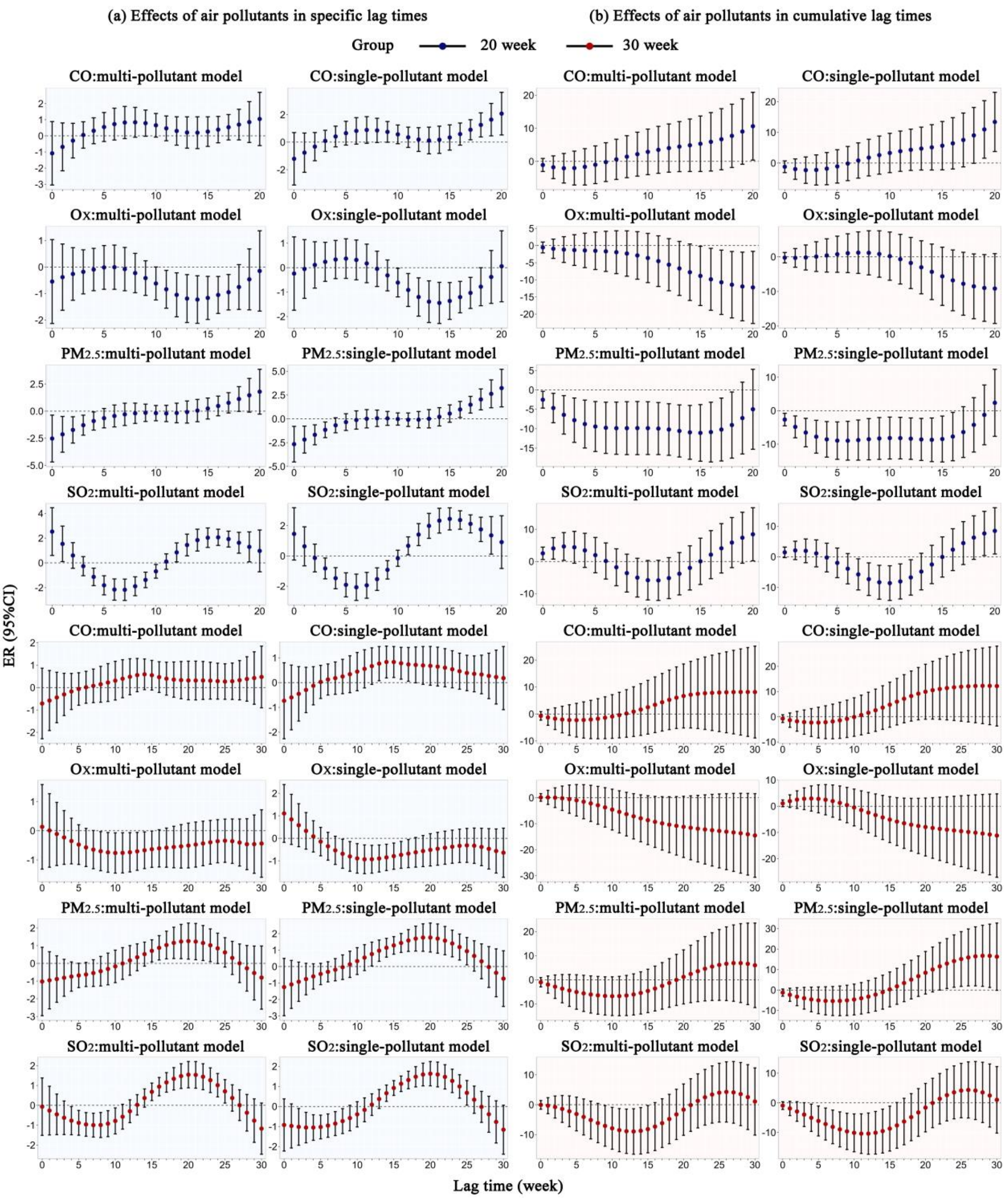

Figure S3. Effects of air pollutants on the risk of pulmonary tuberculosis incidence in specific and cumulative lag times when changing the maximum lag period (20 or 30 weeks).

Abbreviations: $PM_{2.5}$, particulate matter with an aerodynamic diameter of 2.5 μm or less; CO, carbon monoxide; O_X, the combined oxidant capacity; SO_2, Sulfur dioxide.

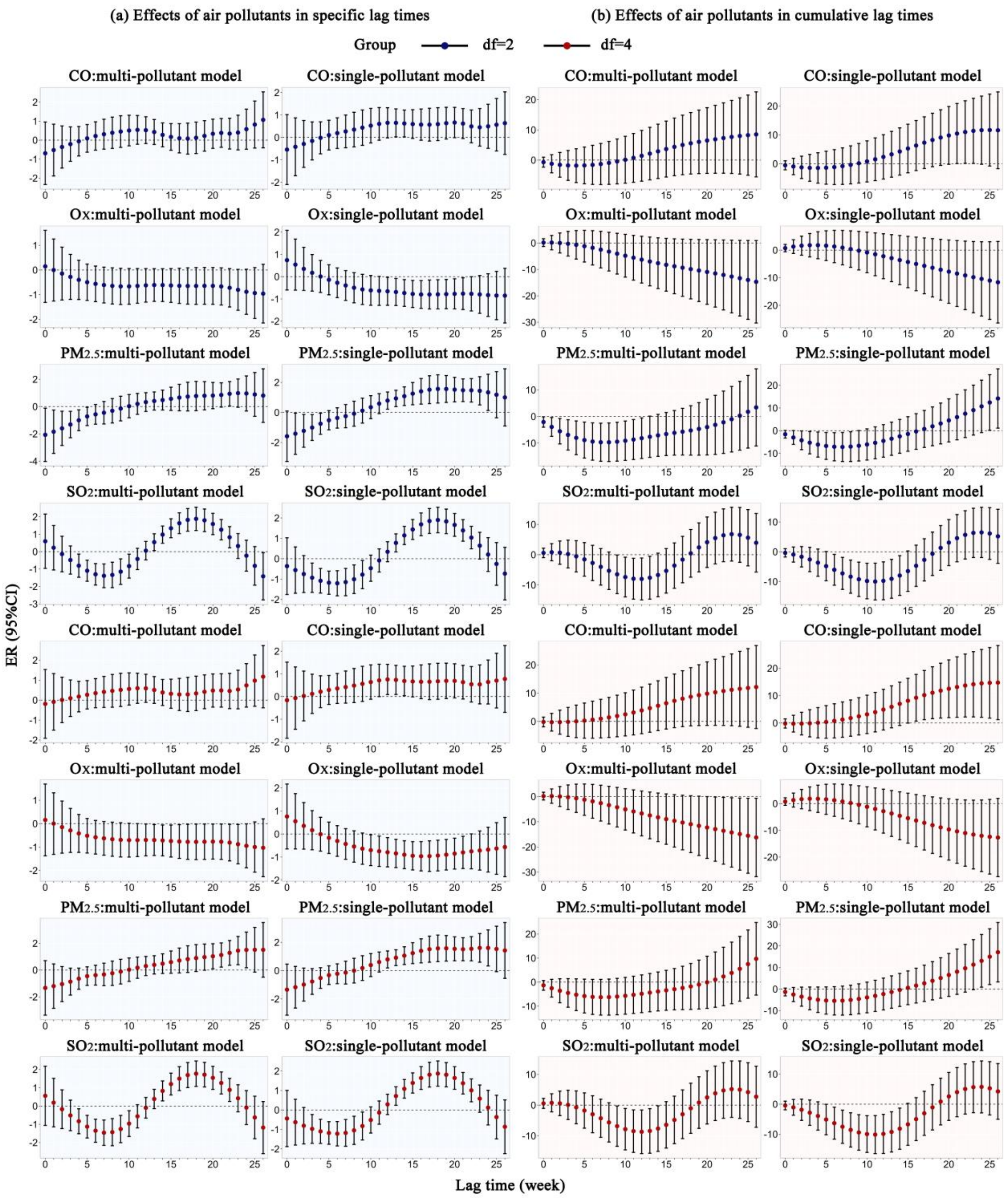

Figure S4. Effects of air pollutants on the risk of pulmonary tuberculosis incidence in specific and cumulative lag times when changing the parameter settings of the degree of freedom (2 or 4).

Abbreviations: $PM_{2.5}$, particulate matter with an aerodynamic diameter of 2.5 μm or less; CO, carbon monoxide; O_X, the combined oxidant capacity; SO_2, Sulfur dioxide.

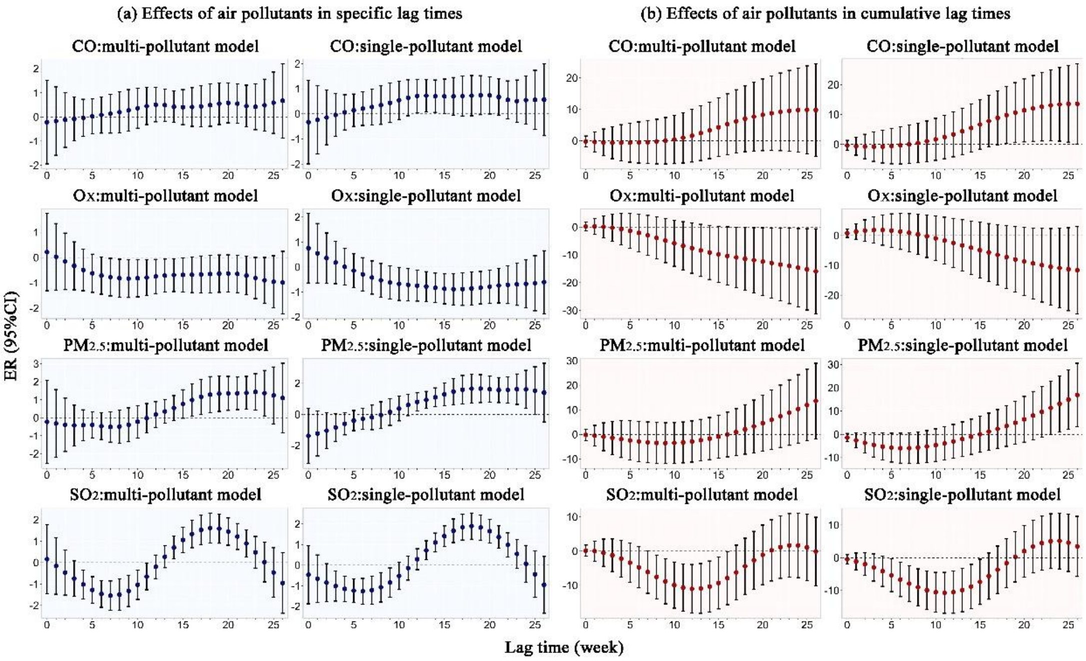

Figure S5. Effects of air pollutants on the risk of pulmonary tuberculosis incidence in specific and cumulative lag times after adjusting for seasonal factors as covariates in the model.

Abbreviations: $PM_{2.5}$, particulate matter with an aerodynamic diameter of 2.5 μm or less; CO, carbon monoxide; O_X, the combined oxidant capacity; SO_2, Sulfur dioxide.
